# Computational Biomarker Pipeline from Discovery to Clinical Implementation: Plasma Proteomic Biomarkers for Cardiac Transplantation

**DOI:** 10.1371/journal.pcbi.1002963

**Published:** 2013-04-04

**Authors:** Gabriela V. Cohen Freue, Anna Meredith, Derek Smith, Axel Bergman, Mayu Sasaki, Karen K. Y. Lam, Zsuzsanna Hollander, Nina Opushneva, Mandeep Takhar, David Lin, Janet Wilson-McManus, Robert Balshaw, Paul A. Keown, Christoph H. Borchers, Bruce McManus, Raymond T. Ng, W. Robert McMaster, for the Biomarkers in Transplantation and the NCE CECR Prevention of Organ Failure Centre of Excellence Teams

**Affiliations:** 1 NCE CECR Prevention of Organ Failure (PROOF) Centre of Excellence, Vancouver, British Columbia, Canada; 2 Department of Statistics, University of British Columbia, Vancouver, British Columbia, Canada; 3 Department of Pathology and Laboratory Medicine, University of British Columbia, Vancouver, British Columbia, Canada; 4 University of Victoria Genome BC Proteomics Centre, Victoria, British Columbia, Canada; 5 Immunity and Infection Research Centre, Vancouver, British Columbia, Canada; 6 Department of Medicine, University of British Columbia, Vancouver, British Columbia, Canada; 7 Immunology Laboratory, Vancouver General Hospital, Vancouver, British Columbia, Canada; 8 UBC James Hogg Research Centre, Vancouver, British Columbia, Canada; 9 Department of Computer Science, University of British Columbia, Vancouver, British Columbia, Canada; 10 Department of Medical Genetics, University of British Columbia, Vancouver, British Columbia, Canada; University of Zurich and Swiss Institute of Bioinformatics, Switzerland

## Abstract

Recent technical advances in the field of quantitative proteomics have stimulated a large number of biomarker discovery studies of various diseases, providing avenues for new treatments and diagnostics. However, inherent challenges have limited the successful translation of candidate biomarkers into clinical use, thus highlighting the need for a robust analytical methodology to transition from biomarker discovery to clinical implementation. We have developed an end-to-end computational proteomic pipeline for biomarkers studies. At the discovery stage, the pipeline emphasizes different aspects of experimental design, appropriate statistical methodologies, and quality assessment of results. At the validation stage, the pipeline focuses on the migration of the results to a platform appropriate for external validation, and the development of a classifier score based on corroborated protein biomarkers. At the last stage towards clinical implementation, the main aims are to develop and validate an assay suitable for clinical deployment, and to calibrate the biomarker classifier using the developed assay. The proposed pipeline was applied to a biomarker study in cardiac transplantation aimed at developing a minimally invasive clinical test to monitor acute rejection. Starting with an untargeted screening of the human plasma proteome, five candidate biomarker proteins were identified. Rejection-regulated proteins reflect cellular and humoral immune responses, acute phase inflammatory pathways, and lipid metabolism biological processes. A multiplex multiple reaction monitoring mass-spectrometry (MRM-MS) assay was developed for the five candidate biomarkers and validated by enzyme-linked immune-sorbent (ELISA) and immunonephelometric assays (INA). A classifier score based on corroborated proteins demonstrated that the developed MRM-MS assay provides an appropriate methodology for an external validation, which is still in progress. Plasma proteomic biomarkers of acute cardiac rejection may offer a relevant post-transplant monitoring tool to effectively guide clinical care. The proposed computational pipeline is highly applicable to a wide range of biomarker proteomic studies.

## Author Summary

Novel proteomic technology has led to the generation of vast amounts of biological data and the identification of numerous potential biomarkers. However, computational approaches to translate this information into knowledge capable of impacting clinical care have been lagging. We propose a computational proteomic pipeline for biomarker studies that is founded on the combination of advanced statistical methodologies. We demonstrate our approach through the analysis of data obtained from heart transplant patients. Heart transplantation is the gold standard treatment for patients with end-stage heart failure, but is complicated by episodes of immune rejection that can adversely impact patient outcomes. Current rejection monitoring approaches are highly invasive, requiring a biopsy of the heart. This work aims to reduce the need for biopsies, and demonstrate the power and utility of computational approaches in proteomic biomarker discovery. Our work utilizes novel high-throughput proteomic technology combined with advanced statistical techniques to identify blood markers that guide the decision as to whether a biopsy is warranted, reduce the number of unnecessary biopsies, and ultimately diagnose the presence of rejection in heart transplant patients. Additionally, the proposed computational methodologies can be applied to a range of proteomic biomarker studies of various diseases and conditions.

## Introduction

After the first successful human-to-human heart transplant in 1967, cardiac transplantation became the primary therapy for patients with end-stage heart failure due to dilated cardiomyopathy or ischemic heart disease. Improvements in immunosuppressive drug therapies have significantly increased the number of successful transplants, yet episodes of acute rejection and progression of chronic rejection remain major factors that negatively impact long term graft survival. Acute rejection is predominantly considered to be an immunological reaction in response to the major and minor histocompatibility antigens recognized as ‘foreign’ by the graft recipient. This process triggers the subsequent activation, migration and infiltration of immune cells such as T- and B-lymphocytes, which can ultimately lead to cellular- and antibody-mediated destruction of the heart allograft tissue [1]. Endomyocardial biopsy (EMB), through which histological features such as cellular infiltration and myocyte damage can be observed, is currently considered to be the only reliable gold standard for diagnosis and monitoring of acute cardiac allograft rejection [2]. However, the invasive and qualitative nature, risk of complications, associated cost and lack of timeliness of the results render the EMB a suboptimal procedure for routine monitoring [3]. A more reliable, minimally invasive, inexpensive, and early diagnostic tool to monitor graft survival remains a significant clinical unmet need.

Since proteins may serve as molecular indicators (i.e., biomarkers) of cardiac allograft rejection, plasma proteomics offers an attractive and promising avenue for the development of diagnosis tools for cardiac transplantation [4]. Technical advances in the field of quantitative proteomics in the last decade have enabled the identification and quantitation of thousands of proteins and have stimulated a large body of research focused on the discovery of new biomarkers. However, the translation of candidate biomarkers from discovery research into proteomic tests for clinical use has faced significant challenges, due mostly to a lack of an adequate analytical pipeline [5], [6], [7], [8]. In a significant step forward, technological proteomic pipelines have recently been proposed, optimizing the design of the discovery, validation, and clinical implementation stages of biomarker studies [8], [9], [10], [11]. Nevertheless, the development of new clinical proteomic tests hinges on a tailored computational pipeline to distill the information contained in thousands of proteins into an accurate classifier score with demonstrable clinical utility.

Computational proteomics is a new and expanding field of research which primarily focuses on data management and mass-spectra analysis for the discovery phase of biomarker studies [12], [13], [14]. Although previous work has acknowledged the need of a tailored computational pipeline in proteomics (e.g.,[15]), a systematic and complete process that specifically addresses the challenges emerging from proteomic studies has not been proposed or demonstrated to date. Using unsuitable methodological tools to explore and analyze the data may result in the selection of biomarkers that ultimately fail in the final stages of validation, or may fail to select relevant biomarkers. For example, identifying a panel of candidate markers based only on the comparison of relative abundance between case and control samples, or the use of classical statistical tests when the sample size of the study is too small, may result in the identification of many false candidate markers.

**Figure 1:**
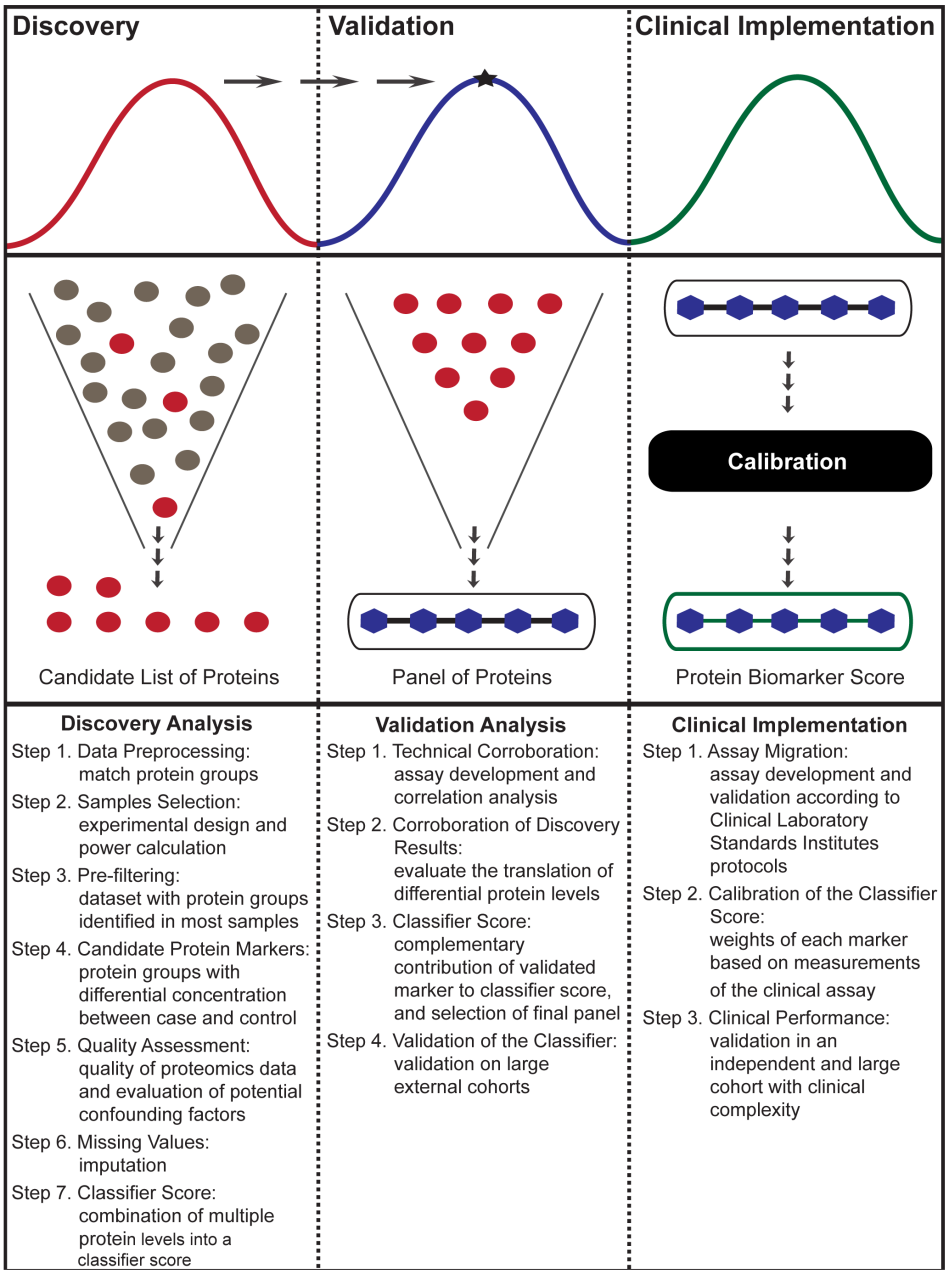
Proteomic computational pipeline synopsis. The 3-stage computational pipeline enables an initial untargeted exploration of the plasma proteome resulting in a list of potential biomarkers, followed by the validation of a set of candidate biomarkers that emphasizes the combination of candidate protein biomarkers into a classifier score with clinical utility. The bottom panel outlines the main steps of the computational pipeline that provide a systematic process from discovery to validation to clinical implementation of plasma protein biomarkers.

We complement previous work by proposing a computation pipeline powered by extensive statistical analysis for all stages of quantitative proteomics biomarker studies (Figure 1). At the discovery stage, the pipeline focuses on selecting an appropriate experimental design and statistical methodologies to identify and assess a panel of candidate biomarkers. At the validation stage, the pipeline emphasizes on the migration of discovery results to the validation platform, and the development and validation of a biomarker classifier. At the clinical implementation stage, the main aims are to develop an assay suitable for clinical deployment, and to calibrate the biomarker classifier using the developed assay.

We demonstrate the power of our methodology in a proteomic biomarker study in the context of cardiac transplantation, with a goal towards the development of a more accurate and less invasive blood test for monitoring graft survival. Our work identified a panel of five candidate plasma proteins that clearly discriminates acute cardiac allograft rejection from non-rejection. These biomarker proteins distribute broadly among three relevant biological processes: cellular and humoral immune responses, acute phase inflammatory pathways and lipid metabolism. Of the five candidate biomarkers, we corroborated four using two independent platforms. A classifier score based on these four corroborated proteins measured by multiple reaction monitoring mass-spectrometry (MRM-MS) demonstrated that plasma protein biomarkers have significant potential in serving as a reliable, minimally-invasive, inexpensive, and timely diagnostic tool for acute cardiac allograft rejection. Our results advance the approaches to diagnosis with respect to cardiac transplantation biomarker, as well as the computational methodologies tailored for a wide range of proteomic biomarker studies.

## Methods

A synopsis of the computational pipeline proposed in this study is illustrated in Figure 1. We first describe in detail each of its steps for the discovery, validation, and clinical implementation stages. We then present a brief description of the materials and methods related to the biomarker study in cardiac transplantation used to illustrate the proposed methodology.

### Computation Pipeline: Discovery Stage

Recent technological advances in quantitative proteomics have enabled the untargeted quantitation and identification of hundreds to thousands of proteins simultaneously from complex samples such as human plasma. The aim of the discovery pipeline is to create a list of candidate markers from an extensive set of proteins identified and measured within each sample.

#### Step 1: Data preprocessing

The first step of the discovery pipeline is to assemble the data generated by (untargeted) quantitative proteomics approaches to perform further statistical analysis. As broadly reviewed by Nesvizhskii and Aebersold [16], the appealing peptide-centric nature of most untargeted proteomics methods is faced with the challenge of recovering the identities of the proteins, originally present in the sample, from the list of peptides identified based on the observed MS/MS spectra. In accordance with established guidelines [17], most software tools report minimal protein lists sufficient to explain all observed peptides using protein groups ([16] [18], and the example in Figure S3). In general, each protein group contains proteins with a high degree of sequence similarity, including multiple entries for one gene product, and isoforms or multiple members of a protein family.

Because for many cases there is insufficient evidence to determine which protein(s) from each group was originally present in the analyzed samples, a comprehensive exploration of the data needs to link and compare protein groups, instead of single protein identities, across multiple experimental runs. Thus, we used an algorithm, called Protein Group Code Algorithm (PGCA), to pre-process protein summaries organized using protein groups (manuscript in preparation). PGCA creates global protein groups from connected groups identified across the different runs and assigns a protein group code (PGC) to each global group. Using this PGC to link groups across multiple runs enables the analysis of interesting proteins that are identified within groups with an unstable composition across runs (further details are given as supporting materials in Text S1).

#### Step 2: Sample selection

Motivated by the diagnosis study in cardiac transplantation, this study was designed to ensure a rigorous case-control analysis at the event time. Thus, the discovery cohort (training set) was constructed by selecting one sample per patient to maintain usual assumptions of independence between samples in statistical tests. Samples in the case and control groups were matched 1 to 2 by time post-transplant and, where possible, age and gender. A power calculation was used to determine the number of samples statistically needed within the case and the control groups (Figure S1A). A different design would be required to identify proteins with differential patterns across time requiring a longitudinal analysis of the data [19], [20].

#### Step 3: Pre-filtering

A common observation in most shotgun proteomic studies is that, even when the same sample is processed multiple times, not all peptides are detected in every experimental run (undersampling) [11], [16], [21]. Undersampling may introduce missing values or outlying protein levels in the resulting proteomics data. Considering undersampling [16], [21], the PGCs detected in at least two-thirds of the experimental runs within each analyzed group were considered for subsequent analyses. Although this approach usually increases the number of analyzed proteins, it also introduces missing values in the data.

#### Step 4: Candidate protein markers

An important step of the computational analysis is the selection of candidate protein markers through univariate statistical tests. It is important to note that low sample variance estimates associated with small sample sizes can increase the number of false positives in many classical tests. Thus, when the study sample size is small, we recommend the use of an empirical Bayes method (eBayes) that estimates protein-specific variances by pooling information from other proteins to construct moderated *t*-tests [22]. Further, a robust variant of eBayes tests can be used which is also less sensitive to outlying observations, usually present in proteomics data [22]. In our biomarker study, robust eBayes tests were used to identify a list of candidate markers (two-sided test, significance level alpha = 0.01). Alternative univariate methods, including *t*-tests and Wilcoxon tests, can be used in larger discovery studies [23]. Considering the inherent variability observed in isobaric tagging for relative and absolute protein quantification (iTRAQ) data and the number of analyzed PGCs, *p* values were not adjusted when pursuing multiple inferences as usually performed in microarray data studies. Although the number of false positives was not controlled by this approach, further corroboration of the identified candidate markers was assessed with two independent platforms.

#### Step 5: Quality assessment

Another step of fundamental importance in any discovery study is quality assessment of the identified markers. Several quality control parameters were examined in accordance with guidelines for proteomic data publication [17], including the qualitative reproducibility of the depletion, the number of peptides used to identify the proteins in each sample, and other parameters to examine the quality of the identification and quantitation of the proteomics data (more details are given as supporting material in Text S1).

The quality of the results was also assessed from a statistical perspective, by examining the potential existence of important confounding factors. As previously demonstrated by Culhane and Quackenbush, the signature of the identified panel may be influenced by the experimental design [24], [25]. Failure to account for potential confounding factors may result in the identification of many false candidate markers that fail to pass the test of validation in an independent cohort. A multivariate ANCOVA approach can be used to test if the simultaneous association between the biomarker panel and the main effect (rejection *versus* non-rejection in our case) remains significant when corrected for potential confounding factors. This test was implemented using the GlobalAncova Bioconductor package (version 3.6.0) [24], [25]. Given that the focus of the pipeline is to identify a panel of proteins that *together* discriminate between case and control samples, using a multivariate test is desirable. Later in the pipeline, we also examined the potential effect of the confounding factors on the classifier score.

#### Step 6: Missing values

As some statistical methodologies do not allow the presence of missing values, the imputation of missing values can be a critical step of the computational pipeline. We used a k-nearest neighbor (kNN) approach [26] to minimize the influence of specific modeling or class information through the imputation. For any missing protein level, the kNN method imputes the weighted average of the protein levels from amongst the k-nearest neighbor proteins available for the corresponding sample. The selection of the k nearest proteins is based on the comparison of protein levels with that of the protein of interest available in other samples [26]. Different imputation methods were previously compared in a similar case study, suggesting that the decision to impute is more important than the choice of the imputation method [27].

#### Step 7: Classifier score

The relative levels of the candidate biomarkers were combined into a single classifier score generated by a Linear Discriminant Analysis (LDA) classifier to demonstrate the joint performance of the identified candidate markers. As LDA seeks a linear combination of markers that best discriminates both groups *together*, the proteomics panel can sometimes achieve a satisfactory classification even when single markers do not clearly differentiate groups (i.e., even though some fold-changes appear relatively small). The score was centered at the LDA cut-off point so that samples with positive and negative scores are classified as cases and controls, respectively. Additional details about multivariate classifiers are given in *Step 3* of the Validation Stage.

Although the GlobalAncova analysis performs a simultaneous global assessment for all candidate PGCs, it does not test the influence of potential confounders in the aggregated classifier score. Thus, we also examined the potential effect of the confounding factors on the LDA score by looking at the Pearson correlation between the score and the potential confounders.

### Computation Pipeline: Validation Stage

As widely discussed in the literature, any list of candidate markers identified in a discovery stage must be validated in a large and independent cohort of patients before its clinical utility assessment. To bridge the gap between discovery and clinical technologies, the validation stage is usually performed in an independent platform which provides a timely and cost-effective approach to measure all samples. To overcome the dependence on antibody availability, we developed an MRM-MS assay to complete the validation stage. However, similar analytical steps would have been taken if another independent platform was used.

#### Step 1: Technical corroboration

The first step is to corroborate that the results from the discovery are successfully translated to the proteomic technologies required in the validation stage. This technical corroboration was first examined by a correlation analysis among the protein levels in common samples measured by different proteomic platforms. To control the influence of outliers in the results, these correlations were estimated using the Spearman correlation coefficient.

#### Step 2: Corroboration of discovery results

The second analytical step is to corroborate that the previously identified differentiation of protein levels between case and control samples is still present when the candidate markers are measured using the new platform(s). The number of samples required for this corroboration needs to be determined based on the estimated variation associated with the new platform (Figure S1B). To allow the comparison of results between the discovery and the validation stages, we recommend using the same statistical test as that used in the discovery, e.g., robust eBayes in our biomarker study. Since the purpose of this corroboration is to test the translation of the results in the new platform, a less stringent statistical cutoff can be used, e.g., two-sided test, significance level alpha = 0.05.

#### Step 3: Development of a classifier score

Despite the general consensus that a panel of biomarkers will be required to classify new samples in a clinical setting, a fundamental analytical step often neglected during the validation stage is the examination of the complementary contribution of each candidate marker to classify new samples [6], [28], [29], [30]. In general, most studies identify long lists of candidate markers that are then examined in isolation from each other to discriminate case from control sample groups.

In this study, different classifiers were built using MRM-MS measurements that sequentially incorporate the corroborated proteins to evaluate their complementary contribution to the classification performance. Although these multivariate classifiers were constructed by Linear Discriminant Analysis (LDA) in our biomarker study, alternative methodologies might be considered, including Support Vector Machines, Elastic Net logistic regression analysis, and Random Forests, among others [23], [28]. LDA assigns a weight to each contributing protein to generate an aggregated score, called the classifier score [31]. The numerical weights of each proteomic marker in the aggregated classifier score thus reflect their contribution to jointly differentiate the case from the control groups. As previously explained, the score was centered at the LDA cut-off point.

The examination of the contribution of each biomarker to the classifier performance can be used to select a final classifier (i.e., a final biomarker panel with the corresponding model) to be tested on an external cohort of patients. Since an independent test cohort was not available at this point, all classification performance measures of the panel were estimated by a stratified 6-fold cross-validation (more details are given as supporting material in Text S1). If available, an intermediate cohort can be used to determine the final classifier and the corresponding threshold to be validated. More importantly, the output of this step is a locked-down model (with a threshold selected) to be validated in an independent cohort.

#### Step 4: Validation of the classifier score

In the last step of the validation stage, the classifier score must be validated in a large external cohort of patients. Common performance measures include sensitivity, specificity, and area under the receiver operating curve (AUC) [32] (definitions are given as supporting material in Text S1). Previous methods have been proposed for the determination of the sample size of this stage [33]. In our biomarker study this stage is still in progress.

### Computation Pipeline: Clinical Implementation

The final translation of proteomic results from the validation to the clinical implementation stage requires careful examination of many factors, including the development of assays suitable for clinical laboratories, considerations from health economics, as well as approval of regulatory agencies (e.g., Food and Drug Administration, Conformité Européenne mark) [8]. From a methodological point of view, the following steps are crucial to complete this last stage.

#### Step 1: Assay migration

A critical next step towards clinical implementation is to develop and validate an assay suitable for clinical deployment according to Clinical Laboratory and Standards Institute's protocols to measure the proteins in the identified panel.

#### Step 2: Calibration of the classifier score

After the assay migration step, the biomarker *classifier score* based on the panel proteins, rather than a simple set of individual proteins, needs to be calibrated using the developed assay. Even though the statistical model to integrate these proteins was locked down in *Step 3* of the Validation stage, new weights that reflect the contribution of each biomarker measured in the new assay to the classifier score must be calculated. This calibration can be performed using either the samples from the training set or those from an intermediate set.

#### Step 3: Clinical performance

Lastly, the classifier needs to be tested in an independent and large cohort with clinical complexity. Different performance measures may be evaluated and emphasized depending on the “fit-to-purpose” of the study, including sensitivity, specificity, negative and/or positive predictive value.

### Additional Methods Related to the Biomarker Study

A brief summary of the materials and methods used in the proteomic biomarker study of cardiac transplantation are outlined here and further details are given as supporting material in Text S1.

#### Ethics statement

This study was approved by the Human Research Ethics Board of the University of British Columbia. All patients enrolled in this study signed consent forms.

#### Study cohorts

A prospective, longitudinal study, approved by the Human Research Ethics Board of the University of British Columbia, was conducted on 63 patients, with signed consent, who received a cardiac transplant at St. Paul's Hospital, Vancouver, British Columbia between March 2005 and February 2008. Of these 63 patients, 44 were included in the acute rejection cohort and were thus part of this study. Patient demographic characteristics are summarized in Table S1.

Pre-transplant and protocol heart tissue biopsies were blindedly reviewed by multiple cardiac pathologists and classified according to the current International Society for Heart and Lung Transplantation (ISHLT) grading scale [2]. Samples graded with an ISHLT Grade ≥2R (multi-foci or diffuse immune cell infiltration with significant associated myocyte damage) were considered significant in the current study for the case group (acute rejection, AR). Samples with ISHLT Grade 0R (normal EMB with no evidence of cellular infiltration) were considered to construct the control group (non-rejection, NR). Mild non-treatable rejections (ISHLT Grade 1R; some cellular infiltrate with limited or absence of myocyte damage; 1R) were excluded from the case-control discovery analysis and were only used as test samples and for the correlation analysis.

#### iTRAQ samples and data processing

An important component of the experimental design for an untargeted platform like iTRAQ is the choice of the reference sample used to ensure interpretable results across different runs [34]. Ideally, this sample should contain all relevant proteins related to the study of interest, thus, pooling some samples from the analyzed groups may be a good option. However, to allow the comparison of the samples in the cardiac study with those of other organs and conditions, a common, pooled normal plasma from 16 healthy individuals was used as a reference sample to run all iTRAQ experiments from different cohorts of the Biomarkers in Transplantation (BiT) initiative. This is consistent with the observations made by Song et al. [35] that consistency of the reference used throughout the entire experiment, including future comparative studies, is more relevant than the actual composition of the reference sample.

All blood samples were processed following rigorously defined standard operating procedures [36], [37]. Briefly, peripheral blood samples were drawn into ethylenediaminetetraacetic acid (EDTA) tubes. Plasma was separated and depleted of the 14 most abundant proteins (albumin, fibrinogen, transferrin, IgG, IgA, IgM, haptoglobin, α_2_-macroglobulin, α_1_-acid glycoprotein, α_1_-antitrypsin, apolipoprotein A-I, apolipoprotein A-II, complement C3, and apolipoprotein B) by immunoaffinity chromatography (GenWay Biotech, San Diego, CA), then trypsin digested and labeled (Applied Biosystems; Foster City, CA). Trypsin peptides from the reference sample were labeled with iTRAQ reagent 114 and peptides from 3 patient samples were randomly labeled with reagents 115, 116 and 117. Spotted peptides were analyzed by a 4800 MALDI TOF/TOF analyzer (Applied Biosystems; Foster City, CA), and MS/MS data were processed using ProteinPilot software v2.0 (Applied Biosystems). Database searching was performed against the International Protein Index (IPI HUMAN v3.39 database) [38]. As recommended by the European Bioinformatics Institute (EBI), the IPI accession numbers of the protein identifiers in our biomarker panel were subsequently updated according to the latest version of UniProtKB database. For each analyzed sample, relative protein levels (ratios of labels 115, 116 and 117 relative to 114, respectively) were estimated for each protein group by ProteinPilot. Protein Group Code Algorithm (PGCA) was used to match protein groups identified by Protein Pilot from different experimental runs (manuscript in preparation).

#### Technical validation

Four out of five markers in the panel were assayed either by enzyme-linked immune-sorbent assay (ELISA), for adiponectin (ADIPOQ, R&D Systems, Minneapolis, MN), factor X (FX, Diapharma, West Chester, OH), and β_2_-microglobulin (B2M, standard clinical laboratory), or by immunonephelometric assay (INA) for serum ceruloplasmin (CP, standard clinical laboratory). ELISA/INA assays were not available for phospholipid transfer protein precursor (PLTP). The same pooled plasma control used for iTRAQ, and patient plasma samples were assayed in duplicate or triplicate. An analogous pooled serum control sample was used for CP. Data from ELISA kits was analyzed on a VersaMax Tunable Microplate Reader (Molecular Devices, Sunnyvale, CA).

#### Multiplex MRM-MS assay development

A multiplex MRM-MS assay was developed for the 5 proteins that constitute the cardiac biomarker panel. MRM-MS ion pairs for 16 selected peptides representing the 5 proteins ( Table S2) and their sensitivities were optimized as previously described [39], [40]. Stable isotopically-labeled peptide standards (SIS peptides) were synthesized using Fmoc chemistry with isotopically- labeled amino acids, [^13^C_6_]Lys or [^13^C_6_^15^N_4_]Arg. The absolute concentrations of each synthetic SIS peptide were determined by amino acid analysis. Further details on sample preparation and MRM-MS data acquisition are given as supporting material in Text S1.

#### Statistical analyses

A detailed description of the statistical methods was described in previous subsections. All the statistical analysis was implemented using R version 2.10.1 [41] with the following packages: limma (version 1.9.6) [42], GlobalAncova from Bioconductor (version 3.6.0) [24], [25], impute (version 1.24.0) [26], and ROCR (version 1.0-4) [43].

## Results

The first two stages of the computational pipeline, discovery and validation, were applied to a biomarker study in cardiac transplantation. An overall schematic of the number of samples, design, and proteomics data used at each stage is summarized in Table 1.

**Table 1:** Proteomics biomarker study schematic.

	Platform	Experimental Design	Cohort	Set of Proteins
***Discovery***				
**Data Processing**	iTRAQ	Reference design	Number of patients = 26 Number of samples = 108 (10 AR, 47 1R, 51 NR) Reference = 16 healthy	924 PGCs, of which 43% were identified based on 2 or more peptides
**Pre-filtering**	iTRAQ	PGCs identified in at least 2/3 of case and control samples	Number of patients/samples = 20 (6 AR, 14 NR)	127 PGCs, of which 98% were identified based on 2 or more peptides
**Candidate Markers**	iTRAQ	Case *versus* control, independent samples	Number of patients/samples = 20 (6 AR, 14 NR)	5 PGCs, of which 100% were identified based on 2 or more peptides
**Relevance of Results**	iTRAQ	Longitudinal representation	Number of patients = 26 Number of samples = 108	Classifier score based on 5 PGCs
***Validation***				
**Technical Corroboration**	ELISA/INA	Independent samples, 25 samples (7 AR, 6 1R, 12 NR) in common with the iTRAQ samples	Number of patients/samples = 43 (13 AR, 12 1R, 18 NR) Reference = 16 healthy	4 proteins available in ELISA or INA
**Technical Corroboration**	MRM-MS	Independent samples, 23 samples (7 AR, 6 1R, 11 NR) in common with the iTRAQ and ELISA samples	Number of patients/samples = 23 (7 AR, 6 1R, 11 NR) Reference = 16 healthy	5 proteins, 16 peptides
**Classifier Development**	ELISA/INA	Case *versus* control, independent samples	Number of patients/samples = 30 (12 AR, 18 NR)	Classifier score based on 4 corroborated proteins
**Classifier Development**	MRM-MS	Case *versus* control, independent samples	Number of patients/samples = 17 (6 AR, 11 NR)	Classifier score based on 4 corroborated proteins

Overall schematic of the cardiac transplantation study following the computational pipeline. PGC = protein group code, AR = acute rejection, 1R = mild non-treatable rejection, NR = non-rejection.

### Discovery Stage

In the discovery stage, multiplexed iTRAQ-LC-MALDI-TOF/TOF mass spectrometry was used to identify and quantitate proteins from 108 depleted plasma samples representing a time course of 20 weeks from the first 26 patients enrolled (Figure S2). These samples were processed in 50 independent iTRAQ runs, including other samples from the heart cohort. In addition, each iTRAQ run included a normal pooled control plasma sample to provide a common reference across multiple runs. A total of 924 protein groups (PGCs) was cumulatively identified from the 50 runs with an average of 273 PGCs within each run.

Following the selection criteria and the power calculation described in the supporting material (Text S1), the first AR samples from 6 (out of 8) AR patients were selected as cases, and samples from 14 (out of 18) NR patients at matching time points were selected as controls (Figure S2A and Table 1). The remaining 88 longitudinally collected iTRAQ samples were used as test samples to initially validate the results at the Discovery stage. Although samples in this test set are part of BiT cohort, none of them were previously used in the training set. As described in Step 3 of the Discovery stage, only those PGCs identified in at least 2/3 of the AR and the NR groups were considered for further analysis. The resulting data consisted of 127 PGCs measured in at least 4 (out of 6) AR patients and 10 (out of 14) NR patients. Of these 127 PGCs, 51 PGCs contained 133 missing values out of a total of 1020 values (i.e., 51 PGCs×20 patients).

A panel of 5 PGCs was identified with significant differential relative concentrations (robust eBayes *p* value<0.01) between AR and NR samples (Tables 2 and 3). This panel consisted of 3 PGCs that were more abundant in AR *versus* NR samples: B2M, F10, and CP, and 2 PGCs that were less abundant: PLTP, and ADIPOQ (Wilcoxon tests are shown in the Table S3).

**Table 2:** Panel of plasma proteins with differential relative levels between acute rejection and non-rejection samples.

PGC	Gene Symbol	*p* value	Fold-Change
6	CP	0.002	+1.28
151	PLTP	0.003	−1.56
188	B2M	0.004	+1.46
84	F10	0.006	+1.27
92	ADIPOQ	0.007	−1.31

Quantitative results of the discovery analysis. For each protein group code (PGC), corresponding genes (Gene Symbol) of all proteins within the groups are shown in the second column. *p* values calculated by the robust eBayes test, and fold-changes with directions (positive sign for proteins more abundant in acute rejection (AR) relative to non-rejection (NR), and negative sign otherwise) are given. The values in these columns correspond to the PGC and not to a particular protein identifier.

**Table 3:** Identification of protein groups in the panel.

PGC	Gene Symbol	IPI Accession	IPI Protein Name	Uniprot	Uniprot Protein Name
6	CP	IPI00017601.1	Ceruloplasmin precursor	Q1L857 P00450 A5PL27	Ceruloplasmin (Ferroxidase; CP protein)
151	PLTP	IPI00643034.2	Isoform 1 of Phospholipid transfer protein precursor	Q53H91 B3KUE5	Phospholipid transfer protein isoform a variant; Phospholipid transfer proteinPhospholipid transfer protein, isoform CRA_c
		IPI00217778.1	Isoform 2 of Phospholipid transfer protein precursor	P55058	Phospholipid transfer protein (Lipid transfer protein II)
		IPI00022733.3	45 kDa protein	P55058	Phospholipid transfer protein
188	B2M	IPI00004656.2	β_2_-microglobulin	P61769	-
		IPI00796379.1	β_2_-microglobulin protein	F5H6I0	Beta-2-microglobulin
		IPI00868938.1	β2-microglobulin	A6XND9	-
84	F10	IPI00019576.1	Coagulation factor X precursor	P00742 Q5JVE7	Coagulation factor X (Stuart factor; Stuart-Prower factor; Coagulation factor X, isoform CRA_a)
		IPI00552633.2	Coagulation factor X	Q5JVE8	-
92	ADIPOQ	IPI00020019.1	Adiponectin precursor	A8K660 Q15848	Adiponectin C1Q and collagen domain containing; (30 kDa adipocyte complement-related protein; Adipocyte complement-related 30 kDa protein)

Accession numbers and protein names from the IPI database have been updated according to UniProt database. Alternative protein names are given in parenthesis.

The quality assessment of the proteomics data demonstrated a strong confidence regarding identified protein identities, wherein 98% of the 127 analyzed PGCs and all 5 PGCs candidate biomarkers were identified based on two or more peptides (Figure S5). Similarly, results showed an overall good coverage and quantitative levels for the analyzed proteins (Table S4). The potential confounding of the results was examined using all available clinical data close to the event time, including daily dose of immunosuppressants, weight, and blood pressure. The GlobalAncova *p* values (Table 4 and Table S5) demonstrate that the simultaneous relative concentrations of the 5 candidate PGCs remained significantly different in the AR group *versus* the NR group (*p* value<0.05) after adjusting for potential confounders. The correlation values in Table 4 show that none of the clinical variables were highly correlated with the LDA classifier score (*r*<0.5). Overall, the results demonstrated that the identification of the biomarker panel was not confounded by other clinical variables available for this study cohort.

**Table 4:** Confounding factors.

Potential Confounders	GlobalAncova *p* value	Correlation with Score	Acute Rejection Mean (SD)	Non-Rejection Mean (SD)
Weight (kg)	0.008	−0.17	74.38 (17.89)	76.76 (29.88)
Systolic blood pressure (mmHg)	0.015	0.34	133.67 (15.71)	122.00 (18.13)
BUN in blood ( mmoI/L)	0.011	−0.43	14.63 (6.12)	11.64 (5.26)
Creatinine in blood (umoI/L)	0.004	−0.42	145.17 (53.83)	125.86 (55.40)
Glucose in blood (mmoI/L)	0.036	−0.35	6.50 (1.99)	6.06 (2.15)
Neutrophil Number in blood (xA9/L)	0.009	−0.02	6.77 (4.58)	6.56 (4.56)
Cyclosporine daily dose (mg)	0.005	−0.18	175.00 (161.25)	167.86 (195.48)
Mycophonelate Mofetil daily dose (mg)	0.007	−0.38	2250.00 (524.40)	1821.43 (540.91)
Prednisone daily dose (mg)	0.013	0.05	10.83 (8.01)	11.79 (6.08)
Tacrolimus daily dose (mg)	0.014	0.41	1.67 (4.08)	4.00 (5.22)

The GlobalAncova analysis evaluates if the panel protein levels remain significantly differentiated between the acute rejection (AR) and the non-rejection (NR) groups after adjusting for potential confounding factors. A *p* value below 0.05 provides evidence of significant differentiation. We use the clinical data available at the time closest to the collection time of the plasma sample measured by iTRAQ (additional potential confounders are shown in the Table S5). The correlation between the value of potential confounders and the LDA classifier score was evaluated using a Pearson correlation coefficient. The last two columns show the mean and standard deviation (SD) of the clinical variables for the 6 AR samples and 14 NR samples in the discovery cohort.

**Figure 2:**
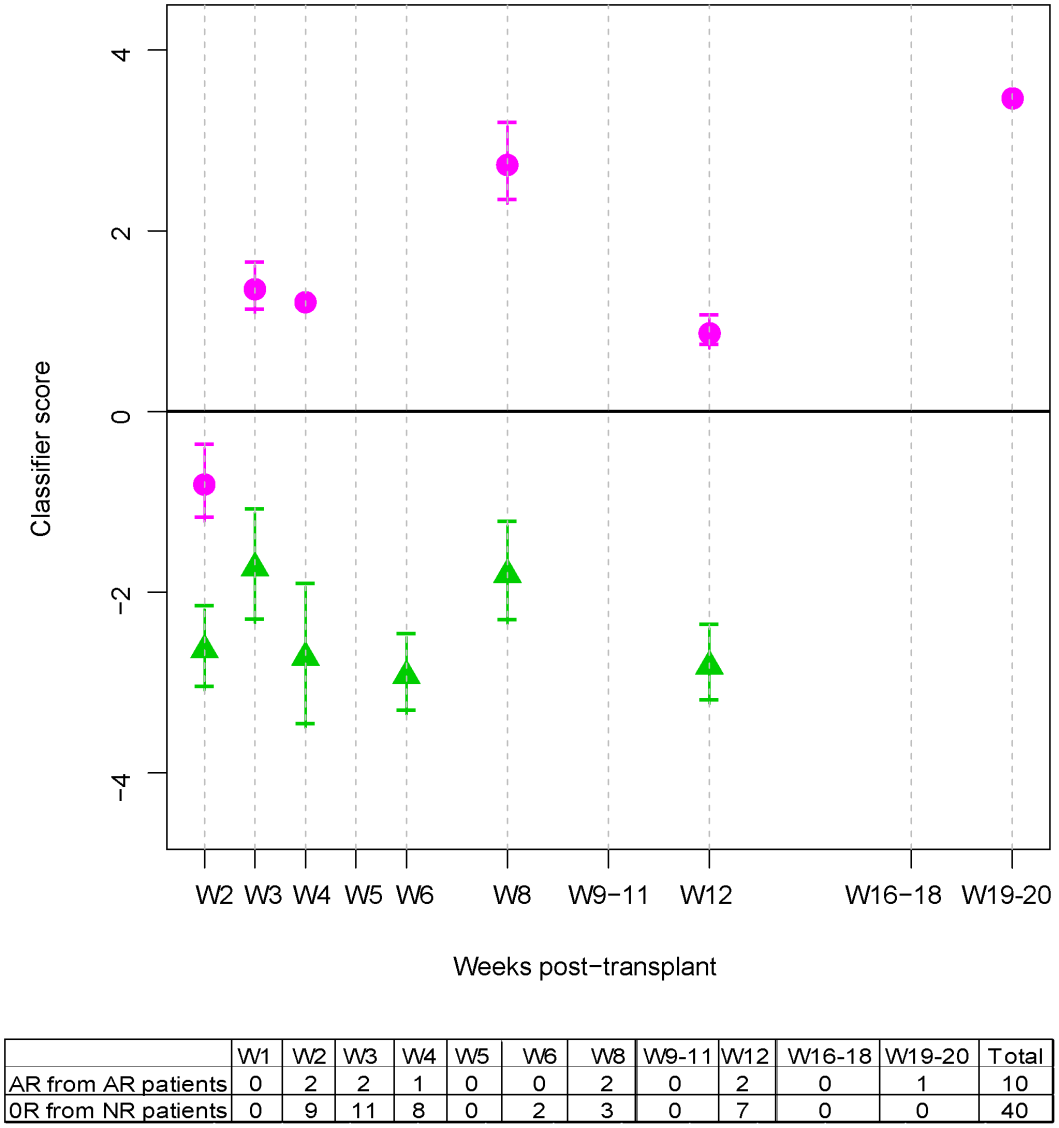
Plasma protein panel. Average Linear Discriminant Analysis classifier score (Classifier score) for all available acute rejection (AR) samples (pink solid point), and non-rejection (NR) samples from NR patients (green solid triangle), at each time point. The score was centered at the LDA cut-off point so that samples with positive and negative scores are classified as “rejection” or “non-rejection”, respectively. Vertical lines represent standard errors. Means and standard error bars can be used to assess differences of the score between groups at any of the studied time points. Sample sizes available at each time point are shown in the bottom table.

To illustrate the joint performance of all candidate markers to discriminate AR from NR samples, the average LDA score was calculated for all the AR samples (n = 10) and the NR samples from NR patients (n = 40) available at each time point (Figure 2). Based on these initial results, the identified candidate markers together discriminated the two groups regardless of which week the rejection occurred after transplantation. Despite this differentiation, the two AR samples in week 2 were still classified as NR (negative score) by the classifier. Although the LDA classifier score was trained to discriminate AR from NR samples, Figure S7 also includes the score of 47 1R mild, non-treatable rejection samples. Average scores of 1R samples from NR patients were in general similar to those of NR samples, while those from AR patients were closer to the average scores of AR samples.

**Figure 3:**
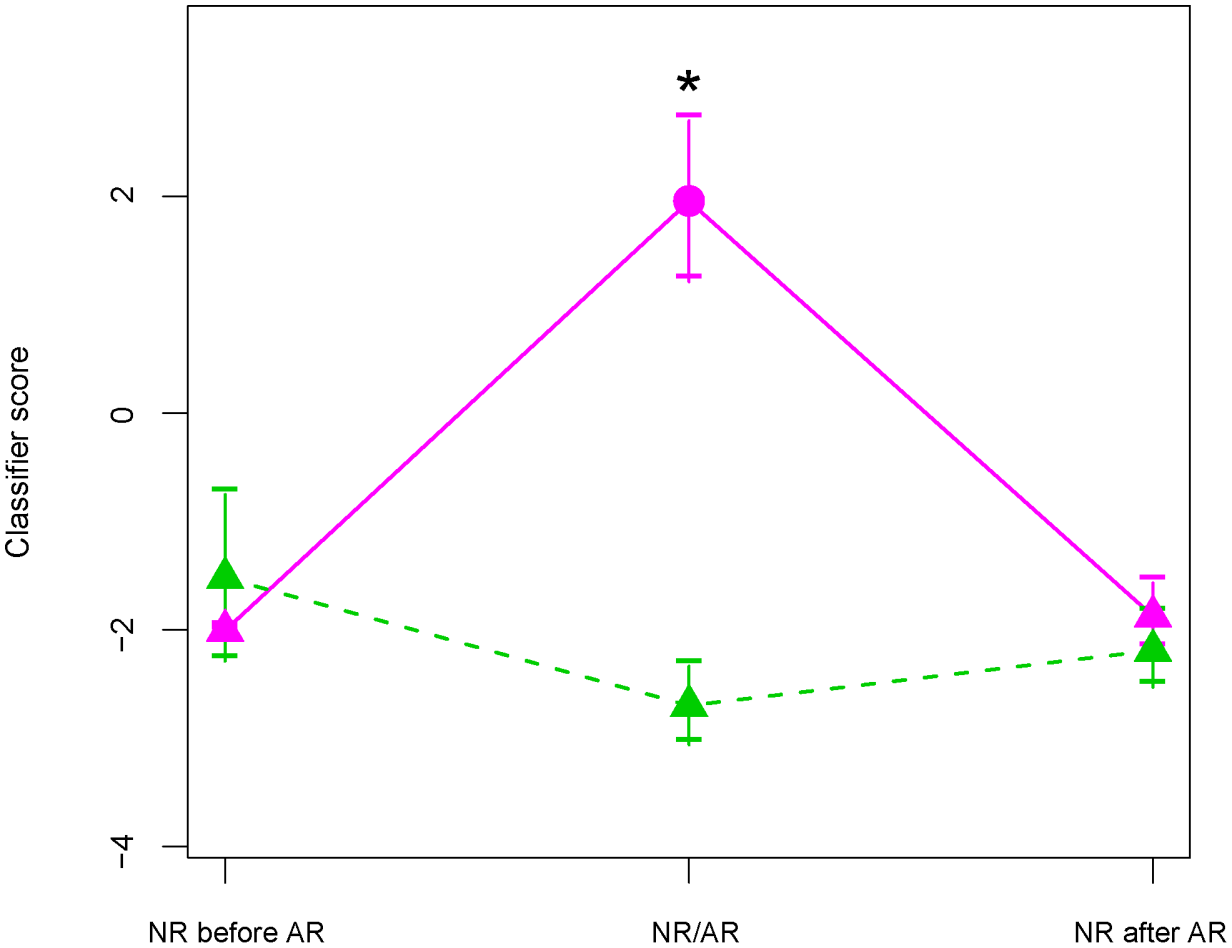
Transition plot. Linear Discriminant Analysis classifier score (Classifier score) when patients transitioned between non-rejection (NR) and acute rejection (AR) episodes. The first consecutive AR time points were averaged (AR, pink solid point) from 7 AR patients. Non-rejection samples from the same patients, before and after AR (“NR before AR” and “NR after AR”) were averaged (pink solid triangle). The average time trend for these samples is represented with a pink solid line. A control curve (dashed green line) was constructed from 9 NR patients matched to AR patients by available time points (green solid triangle). Vertical lines represent standard errors. The asterisk (*) means that the two-sided *t*-test *p* value<0.001.

Figure 3 illustrates the temporal correlation of the score with the diagnosis of rejection. The classifier score for AR patients was at baseline before the rejection episode (pre-rejection point) with a similar average value to that of NR patients at matched time point(s) (no statistical evidence of differentiation). The score for AR patients was differentially elevated at the time point(s) of rejection (as determined by biopsy) compared to that of NR patients (alpha level = 0.05, two-sided t test, *p* value<0.001) at matched time point(s). The score for the AR patients returned to baseline following treatment and resolution of the rejection episode (post-rejection point, non-rejection determined by biopsy) with a similar average value to that of NR patients. In addition, the evaluation of the score across time shows that the biomarker signature is specific to the rejection episodes, rather than reflecting confounded differences or potential bias between the groups (e.g., different medication regimens).

Further results from an initial validation performed in this stage based on 88 test iTRAQ samples not included in the discovery are shown in Figure S6. A total of 3 out the 4 AR and 29 out of the 37 NR samples tested were correctly classified (non-highlighted cells). Similar results were obtained only if a single test sample per patient was randomly selected.

### Validation Stage

The results from the iTRAQ discovery analysis were corroborated and initially validated by two independent assays: ELISA/INA (available for ADIPOQ, F10, B2M, and CP), and MRM-MS (developed for ADIPOQ, F10, B2M, CP, and PLTP). Following the results of the power calculation illustrated in Figure S2B, a total of 43 patients were selected and plasma and serum samples were processed by ELISA/INA for an initial validation cohort that extends the discovery cohort. A subset of 25 of these 43 samples, 7 AR, 6 1R and 12 NR, were also part of the iTRAQ discovery cohort. Of these 25 samples, 23 were also processed by MRM-MS (Table 1 and Figures S2A and S2B). Samples measured by the three assays were used to perform the correlation analysis.

**Figure 4:**
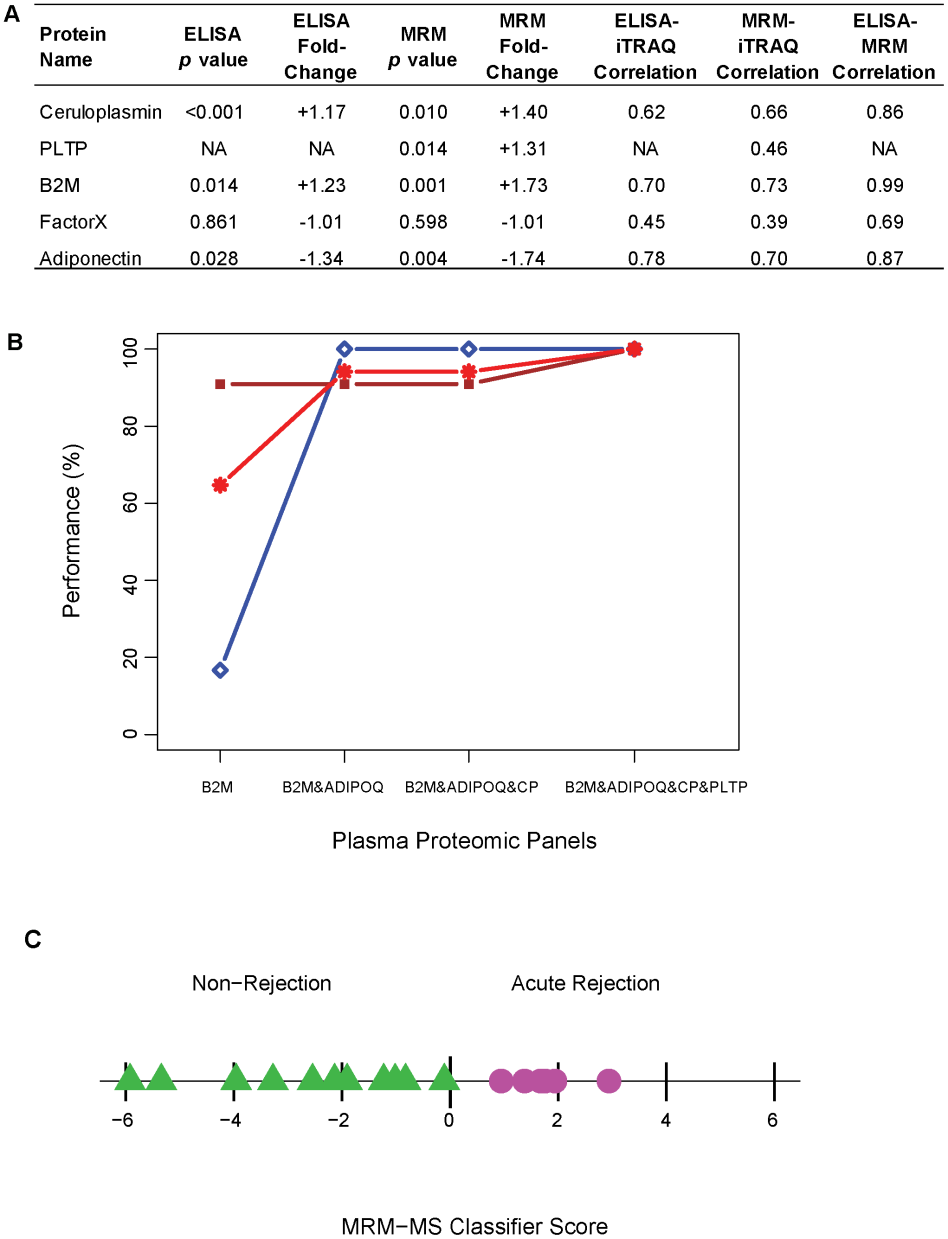
Technical validation. **A.** For both ELISA/INA and MRM-MS corroboration analysis, *p* values were calculated by robust eBayes (12 acute rejection (AR) *versus* 18 non-rejection (NR) samples in ELISA/INA, and 6 AR *versus* 11 NR in MRM-MS, two-sided test). The correlations among platforms are based on all available common samples, i.e., 25 samples measured by iTRAQ and ELISA/INA, and 23 samples measured by iTRAQ, ELISA/INA, and MRM-MS (Figure S2). **B.** Validation performance (y-axis) estimated by a 6-fold cross-validation: sensitivity (blue diamond), specificity (brown square), and area under the receiver operating curve (AUC) (red star) for incremental classifier panels. The sensitivity and specificity estimates were calculated using a probability cut-off of 0.5. The x-axis shows three nested classifier panels based on a single candidate marker (B2M), 2 markers (B2M&ADIPOQ) and 3 markers (B2M&ADIPOQ&CP), respectively, measured by MRM-MS. As F10 was not validated in either ELISA/INA or MRM-MS, it was not included in any MRM-based classifier. **C.** MRM-MS classifier score generated by a 6-fold cross-validation using Linear Discriminant Analysis. Samples with a positive proteomic classifier score are classified as “rejection” and those with a negative score are classified as “non-rejection”.

Results showed good levels of correlations for B2M, ADIPOQ, and CP (r>0.6, Figure 4-A). F10 measurements from both ELISA/INA and MRM-MS and PLTP measurements from MRM-MS did not show a similar degree of correspondence with iTRAQ as seen for other proteins. However, a good correlation was observed between ELISA/INA and MRM-MS for F10 measurements (r = 0.69, Figure 4A).

The differential protein levels between AR and NR samples observed in the discovery stage were successfully translated for 3 of 4 proteins measured by ELISA/INA (B2M, ADIPOQ, and CP, *p* value<0.05), and 4 of 5 proteins measured by MRM-MS (B2M, ADIPOQ, CP, and PLTP) (Figure 4A). Results from the ELISA/INA data provided additional validation in 12 new patients using a platform other than iTRAQ (Table 1, and 0R(E) and 2R(E) samples in Figure S2A). Taken together, with the exception of F10, the results showed that measurements from the three platforms were strongly correlated and corroborated most of the results from the iTRAQ discovery stage.

Figure 4B demonstrates the gain in classification performance by a panel of markers combined together into a multivariate classifier score. Although estimated on a small cohort, the sensitivity estimates improved from 17% for the classifier based only on B2M to 100% for the classifier based on the 4 corroborated protein panel (B2M&ADIPOQ&CP&PLTP), the specificity improved from 91% to 100%, and the AUC improved from 0.25 to the maximum of 1. Based on the classification performance of the evaluated MRM-MS classifier scores, a panel of 4 proteins (B2M, ADIPOQ, CP, and PLTP) was selected to complete the validation stage.

Figure 4C illustrates the resulting classifier score based on the 4-protein panel for the test samples resulting from a 6-fold cross-validation. Samples with a positive proteomic classifier score were classified as “rejection”, and those with a negative score were classified as “non-rejection”. In this initial validation, all test samples were correctly classified by the proteomic classifier score. However, because the test samples in the cross-validation were still part of the discovery analysis, these performance measures cannot be used to characterize the identified classifier. Although similar results were obtained using the ELISA/INA measurements on an extended cohort of patients (Figure S8B), a larger validation in an external cohort of patients is still required to complete this phase. A prospective clinical assessment of the value of these proteomic markers of acute rejection is currently underway using MRM-MS measurements of over 200 samples from six Canadian sites.

## Discussion

Over the last two decades, the accelerating pace of technological progress has initiated a new era in the field of clinical proteomics. In particular, plasma proteomics offers a powerful tool to examine the underlying mechanisms of various diseases and opens novel avenues for biomarkers discoveries. To date, the number and quality of technical resources available for proteomic biomarker studies are well recognized. However, the development of statistical methods to address the challenges that have arisen in the field has lagged behind, dramatically reducing the pace, quality and precision of biomarker studies. An important piece of the puzzle in clinical proteomics is to distill the information contained in the very rich data generated by new proteomic technologies via a tailored computational pipeline [15].

In this study we propose and apply a computational pipeline that provides a systematic process to analyze proteomics data related to biomarker studies. The computational steps described in this study are consistent with, and complement those described in previous technological and analytical pipelines [6], [8], [9], [10]. Our pipeline successfully generated an accurate classifier score based on four plasma proteins to diagnose acute rejection in patients who have received cardiac transplants.

There are likely additional plasma protein biomarkers that were not identified by this approach. For example, additional candidate markers may be identified using a different reference sample or an alternative proteomics platform. However, initial validation results indicate that a classifier score based on 4 corroborated biomarkers can achieve a satisfactory classification of acute rejection and non-rejection samples. If validated in a larger and external cohort of patients, the identified proteomic biomarker panel can be used to develop a more accurate and minimally invasive clinical blood test to monitor allograft rejection.

The analysis may also reveal some biomarkers previously associated with unrelated disease phenotypes, or that are not linked to cardiac transplantation. In general, looking at injury controls is a good idea and ideally one would want to include such to show that the identified panel is specific for the disease of interest. However, such comparisons would require additional carefully phenotyped cohorts, analyzed with the same analytical and technological methods on the same sample source, which for many relevant injuries are difficult or impossible to obtain. The data shown in our study do not address this point and much more work needs to be done on the comparison of acute rejection with other injuries.

The complex pathobiology of acute cardiac allograft rejection is reflected in the heterogeneity of markers identified in this study. The majority of proteins identified distribute broadly among three biological processes, consistent with the current understanding and pathogenesis of acute rejection: cellular and humoral immune responses, acute phase inflammatory pathways and lipid metabolism. Our results also highlight the anticipated distinction between the plasma proteome and that observed in tissue-based discovery studies [44]. As well, while circulating protein markers of acute allograft rejection found by others were mainly indicative of tissue damage and stress [44], we also identified markers that implicate immune and vascular processes, in addition to other aspects of the rejection process. In general, knowing the biological process of the identified markers may lead to a better understanding of disease pathogenesis, and to novel therapeutic targets.

### Cellular and Humoral Immunity

Transplantation elicits a host immune response that encompasses both cellular and humoral immunity, which together lead to graft tissue damage, and episodes of acute and chronic rejection. B2M is a protein associated with MHC Class I histocompatibility antigens, with increased levels reflecting allograft rejection, autoimmune or lymphoproliferative diseases as a result of increased immune activation [45]. Several studies have reported higher circulating levels of B2M in cardiac or renal allograft rejection [46], [47], [48], consistent with our observations. Importantly, our data demonstrates improved classification performance when additional markers are used with B2M.

### Acute Phase Response/Inflammation

Acute rejection resulting from cellular infiltration of the graft leads to severe local inflammation, which has systemic consequences with a concomitant increase in circulating inflammatory markers. The acute phase response to inflammatory stimuli involves the production and release of numerous plasma proteins by the liver. CP, significantly up-regulated in AR relative to NR samples in this study, is a positive acute phase reactant. It is elevated in acute and chronic inflammatory states and elevated plasma CP is also associated with increased cardiovascular disease risk [49]. CP is a player in inflammation, coagulation, angiogenesis, and vasculopathy, but its role in the pathogenesis of acute rejection is unknown. Current evidence supports a relationship between inflammation and coagulation [50]. FX, a key mediator in the conversion of prothrombin to thrombin, is up-regulated in our acute rejection cohort, and this finding may reflect an intersection between inflammatory and coagulation responses in acute rejection. However, this protein was not validated in our study. C reactive protein (CRP), an acute phase reactant protein previously studied in the context of acute cardiac allograft rejection, was not identified in our study. Consistent with this finding, previous work has demonstrated conflicting evidence regarding the informative value of CRP in monitoring acute cardiac allograft rejection [51].

### Lipid Metabolism

Dyslipidemia as a consequence of immunosuppressive therapy has been reported in cardiac allograft recipients, and is a risk factor for chronic rejection [52]. Lipid metabolism is represented by two proteins in our panel: ADIPOQ and PLTP. ADIPOQ is a circulating plasma protein involved in metabolic processes shown to play a role in atherosclerotic cardiovascular diseases [53]. Work by Nakano and others described elevated ADIPOQ as reflective of tolerance following a rat model of orthotopic liver transplantation, suggesting a mechanistic role for this protein and corresponding with the observed decrease in ADIPOQ levels during acute rejection episodes [54]. Further, recent work by Okamoto and colleagues [55] has demonstrated that ADIPOQ inhibits allograft rejection in a murine model of cardiac transplantation. PLTP plays a role in HDL remodeling and cholesterol metabolism but its involvement in acute rejection is unknown.

A comparison between the current panel identified for the diagnosis of cardiac allograft rejection, and that of renal allograft rejection [37], reveals that the biological roles of identified proteins are shared in the setting of both transplantation situations. Moreover, the relative regulation of proteins involved in these biological processes is likewise shared. Our current data reveals a differentiation of particular molecules involved in the pathogenesis of cardiac versus renal allograft rejection.

The plasma protein markers identified in this study have the potential to be further assessed in combinatorial analyses with Biomarkers in Transplantation (BiT) genomic and metabolomic data. Notably, numerous research groups, including the BiT group, have identified potential gene expression markers of cardiac allograft rejection using microarray and qPCR analyses of peripheral and whole blood [56], [57], [58], [59]. These studies provide an opportunity for a systems biology approach to understanding allograft rejection.

Taken together, the panel of protein markers identified and initially validated in this study offers a fresh approach to the diagnosis of acute cardiac rejection, providing novel avenues of investigation and potential new targets for therapeutic intervention. The computational pipeline proposed and applied in this biomarker is highly applicable to a wide range of biomarker proteomic studies.

## Supporting Information

Figure S1 **Power calculations.** **A.** Power curves to design the discovery iTRAQ study, based on an estimated coefficient of variation of 0.25 for iTRAQ relative ratios (in log scale). The sample size of the NR group was assumed to be twice as large as that of the AR group. The red, green and blue curves correspond to fold-changes (ratios of means protein relative levels) of 1.15, 1.2, and 1.3, respectively. **B.** Power curves for the identified markers to be validated by ELISA/INA. The calculation was based on the coefficient of variations and the fold-changes (right table) computed from pilot data. The solid, dotted, and dashed lines correspond to estimates based on ADIPOQ, CP, and B2M data, respectively.
(PDF)

Figure S2 **Study design.** **A.** The 3 boxes at the top show the number of patients and samples in the different cohorts. Some of these patient samples were processed by more than one platform to study the correlation between measurements from different technologies. The table shows the ISHLT Grades of rejections and longitudinal distribution of samples processed with iTRAQ (I), ELISA (E) and/or MRM-MS (M). Filled grey cells indicate samples used in the training set of the iTRAQ discovery analysis. Samples graded with an ISHLT Grade ≥2R (multi-foci or diffuse immune cell infiltration with significant associated myocyte damage) were considered to construct the case group (AR, acute rejection). Samples with ISHLT Grade 0R (normal EMB with no evidence of cellular infiltration) were considered for the non-rejection (NR) control group. Mild non-treatable rejections (ISHLT Grade 1R; some cellular infiltrate with limited or absence of myocyte damage; 1R) were only used as test samples. Asterisks indicate patients with additional complications (e.g., prolonged peri-transplant ischemia, infection, second transplant, etc). Black triangle represents AR biopsy with no plasma sample available. **B.** Overlap of samples in the iTRAQ, the ELISA/INA, and the MRM-MS cohorts. A subset of these samples were included in the training sets of the discovery and corroboration analyses (highlighted cells in panel **A** of this ).
(PDF)

Figure S3 **ProteinPilot's local groups, related protein sequences, and identified peptides.** **A.** Example of the protein group corresponding to β_2_-microglobulin (B2M) as shown in the protein summaries given by ProteinPilot for three distinct experimental iTRAQ runs (ExpID) in the cardiac biomarker study. The Unused, Total and %Cov are identification quality parameters given by ProteinPilot. Quantitative values corresponding to only one of the 3 ratios is shown (115∶114). **B.** Aligned protein sequences from the B2M protein group. Peptides identified by Paragon Software within each experimental iTRAQ runs are shown in bold-black fonts. The accession number chosen by Pro Group Algorithm to represent the group in the protein and peptide summaries (top-identifier) is shown in bold-black font.
(PDF)

Figure S4 **Quality of depletion.** Qualitative reproducibility of the depletion measured on 19 iTRAQ runs used to process all samples in our discovery analysis on 9 of the 14 depleted proteins. At least one AR sample was processed in the first 6 iTRAQ runs and one 0R sample in the last 14 runs. Bars represent average percentages of remaining peptides from depleted proteins in AR (red bar) and in NR (black diagonals) samples. Standard errors are shown with vertical lines.
(PDF)

Figure S5 **Proportion of protein group codes (PGC's) identified using different peptide counts (** ***p*** **).** Peptide counts used to identify each PGC differ run to run. Thus, average peptide counts across iTRAQ runs were used for PGC's identified in multiple runs. “Total”, “Analyzed” and “Panel” represent the sets of PGC's detected in at least one of the 18 samples included in the discovery, detected in at least 2/3 of the AR and NR groups, and identified with significant differential relative concentrations, respectively. Each bar represents the proportion of PGC's within each group identified based on *p* distinct peptides.
(PDF)

Figure S6 **Classification results.** Set of AR and NR samples in the test set classified based on the LDA score. Samples used in the discovery and 1R samples were not included in the test set. Biopsy and classifier results are shown in the top-left and bottom-right corners of each cell, respectively. For example, the second week sample (W2) of the first acute rejection patient (AR1) was classified as AR based on the biopsy (top-left) and as a 0R based on the proteomic classifier (bottom-right). Misclassified samples are highlighted with filled bold-font cells.
(PDF)

Figure S7 **Classifier score for 1R samples.** Average score generated by LDA for all available AR samples (pink solid dot), 1R samples from AR patients (pink open dot), 1R samples from NR patients (green open triangle), and NR samples from NR patients (green solid triangle), at each time point. Standard errors are represented with vertical lines. Sample sizes available at each time point are shown in the table.
(PDF)

Figure S8 **Technical validation.** **A.** Scatter plots of protein concentrations (y-axis) for 13 AR *versus* 18 NR samples (x-axis) in ELISA/INA, and 6 AR *versus* 11 NR in MRM-MS for the validated proteins. Median values are represented by horizontal lines within each group. **B.** Classifiers performance (y-axis) estimated by a cross-validation: Sensitivity (solid dot), specificity (solid triangle), and accuracy (open square) for incremental classifier panels. The x-axis shows three nested classifier panels based on a single marker (B2M), 2 markers (B2M&ADIPOQ) and 3 markers (B2M&ADIPOQ&CP), respectively, measured by ELISA/INA. As F10 and PLTP were not validated in ELISA/INA they were not included in any ELISA/INA-based classifier.
(PDF)

Table S1 **Demographic characteristics of patients.** Numbers in parentheses are percentages unless otherwise stated.
(PDF)

Table S2 **MRM-MS assay.** The “Component Name” column shows the protein group code (PGC), the peptide sequence, and the precursor/product ion pairs. The last column shows the retention times used to acquire the MRM-MS data.
(XLSX)

Table S3 **Corroboration of the discovery results by a different statistical test.** Comparison of *p* values calculated by the robust eBayes test (fifth column) used in the iTRAQ discovery and the Wilcoxon test (sixth column). Tests were based on iTRAQ data for the 5 identified candidate markers (6 AR *versus* 14 NR samples).
(PDF)

Table S4 **Quality control parameters of proteomic data.** “Unused” represents the median of the Unused ProtScores calculated by ProteinPilot for the top protein within each iTRAQ run protein group. Unused values equal to 2.0 is equivalent to a 99% confidence. Similarly, “Coverage” and “Error factor” represent the median of percent coverage and error factor measures calculated by ProteinPilot for each group in each iTRAQ run. “Peptide count” shows the average of unique peptide counts, excluding miscleavages, used for protein identification and quantitation by ProteinPilot in each iTRAQ run. “Missing AR/NR” shows the number of samples in the rejection (AR) and non-rejection (NR) groups in which each protein group was not detected. “Length” and “pI/molecular mass” contain the number of amino acids in each sequence and the isoelectric point/molecular mass (kDa) for each protein, respectively. ^a^Values in these columns correspond to the PGC and not to a particular protein identifier.
(PDF)

Table S5 **Confounding factors.** The GlobalAncova analysis evaluates if the panel protein levels remain significantly differentiated between the acute rejection (AR) and the non-rejection (NR) groups after adjusting for potential confounding factors. A *p* value below 0.05 provides evidence of significant differentiation. We use all clinical data available at the time closest to the collection time of the plasma sample measured by iTRAQ. The correlation between the value of potential confounders and the LDA classifier score was evaluated using a Pearson correlation coefficient. The last two columns show the mean and standard deviation (SD) of the clinical variables for the 6 AR samples and 14 NR samples in the discovery cohort.
(PDF)

Text S1 **A detailed description of sample selection criteria, plasma collection, depletion, trypsin digestion, iTRAQ labeling, 2D-LC chromatography, mass spectrometry, data processing procedures and analyses are given in this supporting material.**
(PDF)
