# Supplementary figures and images for: Computational Biomarker Pipeline from Discovery to Clinical Implementation: Plasma Proteomic Biomarkers for Cardiac Transplantation
Source: PLoS Comput Biol. 2013 Apr 4;9(4):e1002963. doi: 10.1371/journal.pcbi.1002963 (PMC3617196; doi:10.1371/journal.pcbi.1002963)

A

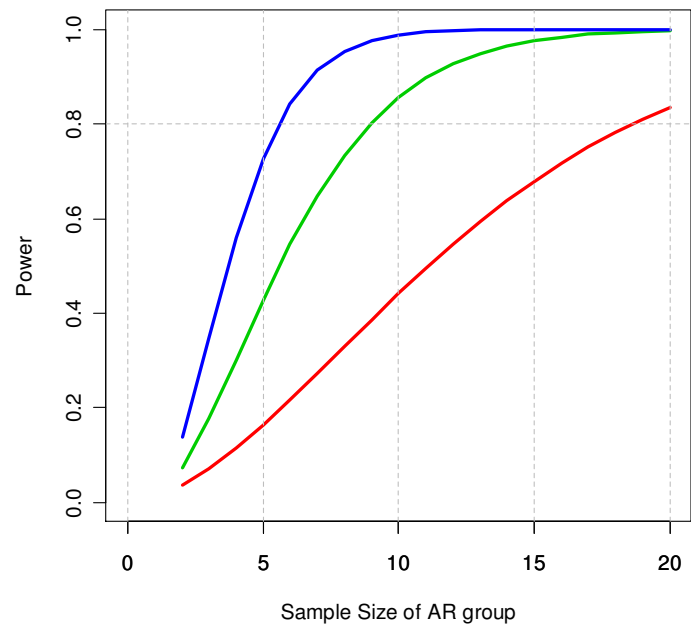

B

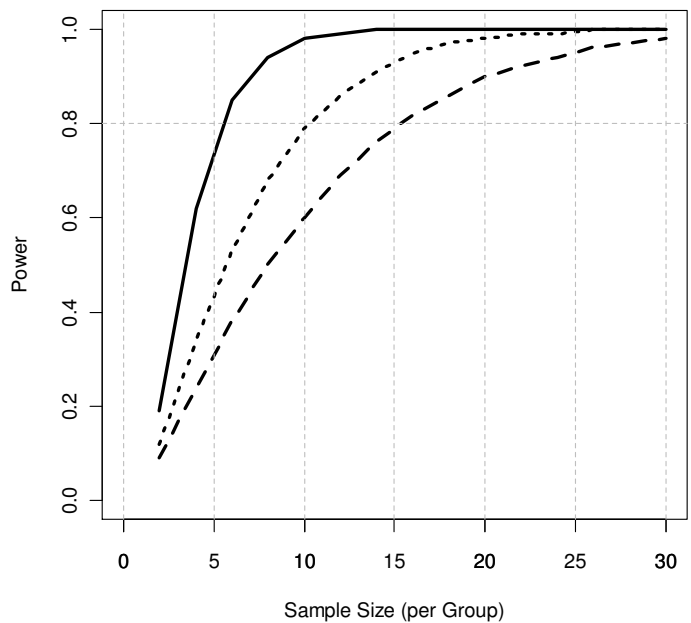

| Protein Name  | Fold-Change | Coefficient of Variation |
|---------------|-------------|--------------------------|
| Adiponectin   | 2.60        | 0.50                     |
| B2M           | 1.57        | 0.43                     |
| Ceruloplasmin | 1.24        | 0.17                     |
| FactorX       | 1.05        | 0.10                     |

Supplement: Figure S1 — Power calculations. A. Power curves to design the discovery iTRAQ study, based on an estimated coefficient of variation of 0.25 for iTRAQ relative ratios (in log scale). The sample size of the NR group was assumed to be twice as large as that of the AR group. The red, green and blue curves correspond to fold-changes (ratios of means protein relative levels) of 1.15, 1.2, and 1.3, respectively. B. Power curves for the identified markers to be validated by ELISA/INA. The calculation was based on the coefficient of variations and the fold-changes (right table) computed from pilot data. The solid, dotted, and dashed lines correspond to estimates based on ADIPOQ, CP, and B2M data, respectively. (PDF) [file pcbi.1002963.s001.pdf]

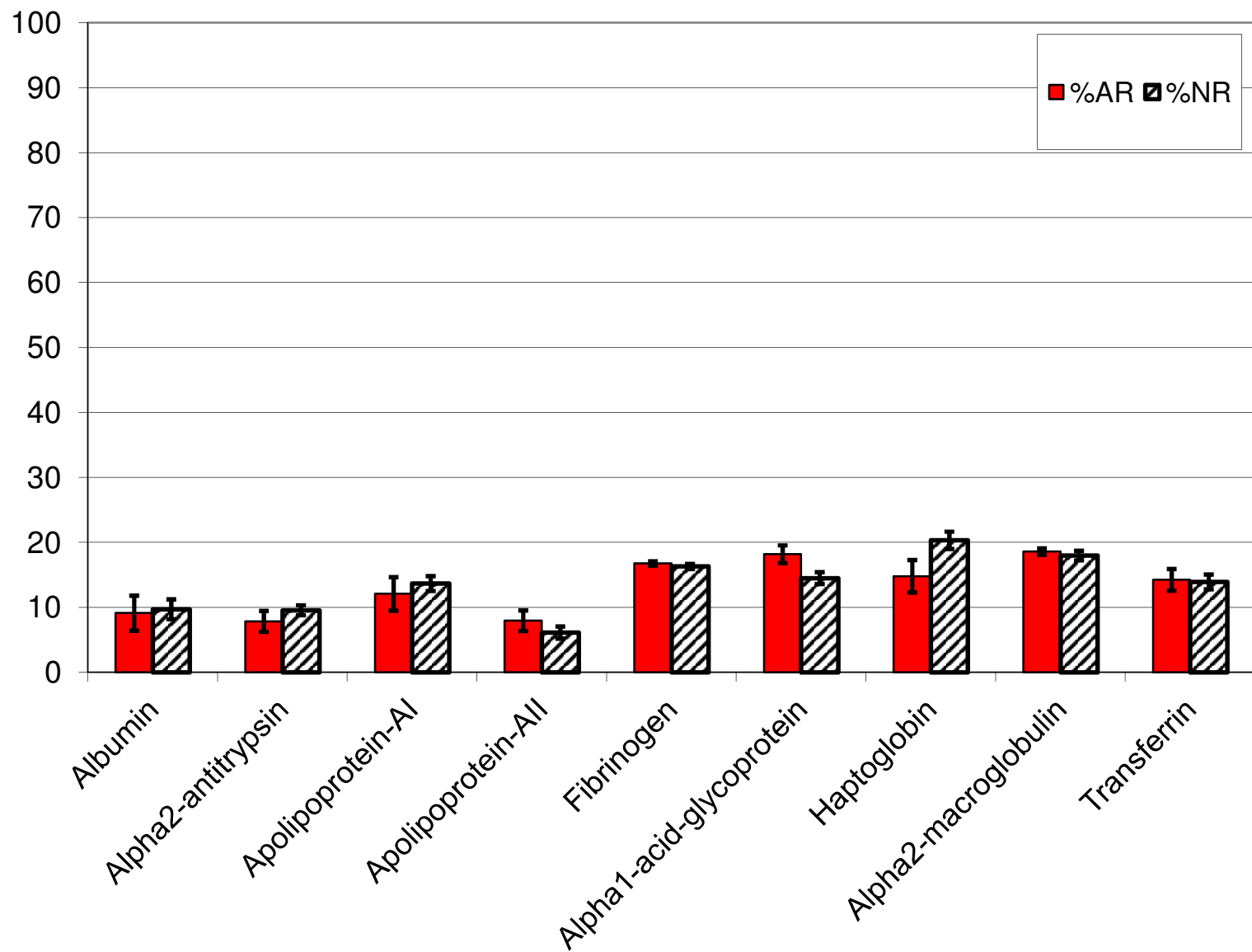

Supplement: Figure S4 — Quality of depletion. Qualitative reproducibility of the depletion measured on 19 iTRAQ runs used to process all samples in our discovery analysis on 9 of the 14 depleted proteins. At least one AR sample was processed in the first 6 iTRAQ runs and one 0R sample in the last 14 runs. Bars represent average percentages of remaining peptides from depleted proteins in AR (red bar) and in NR (black diagonals) samples. Standard errors are shown with vertical lines. (PDF) [file pcbi.1002963.s004.pdf]

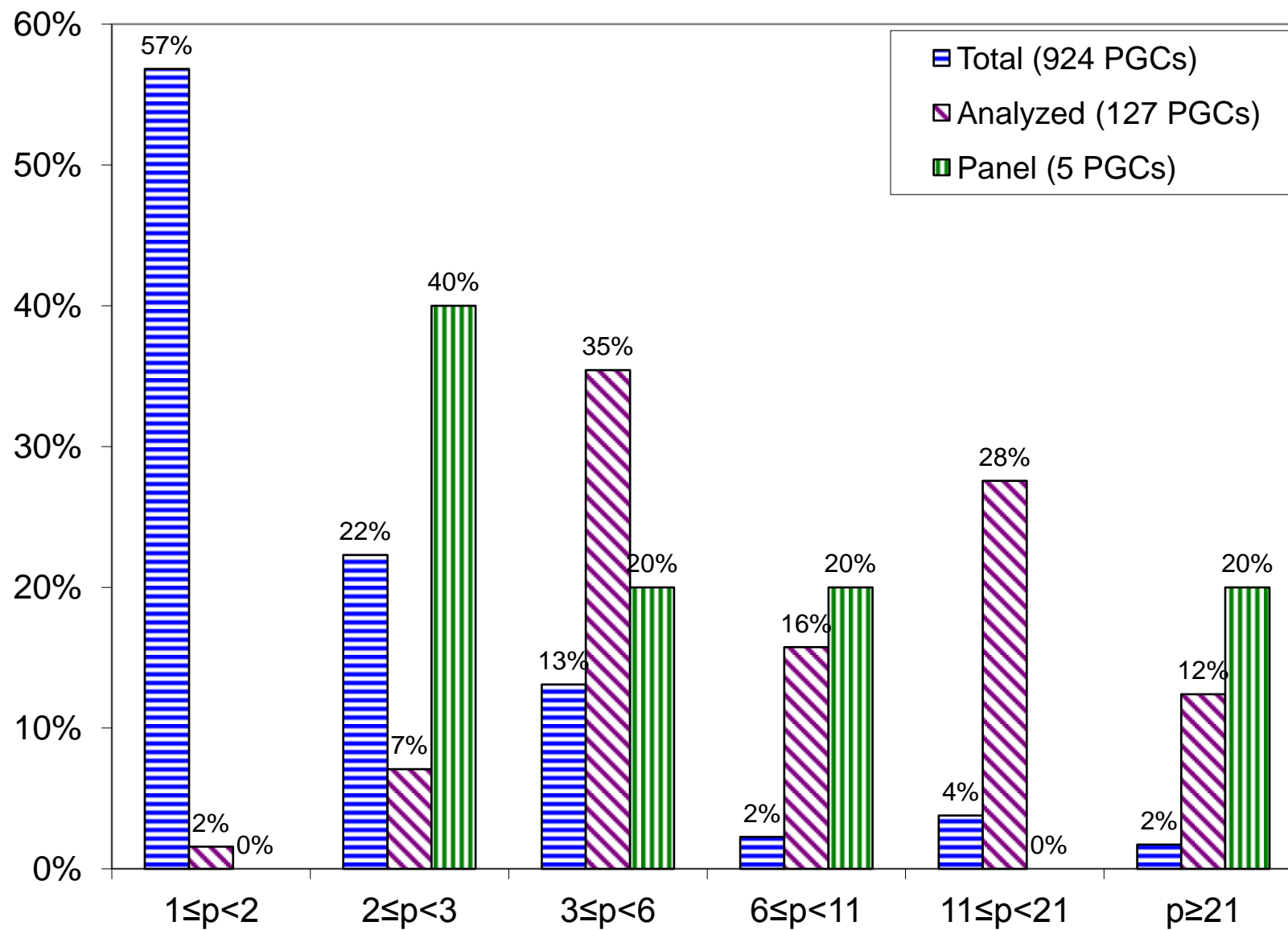

Supplement: Figure S5 — Proportion of protein group codes (PGC's) identified using different peptide counts ( p ). Peptide counts used to identify each PGC differ run to run. Thus, average peptide counts across iTRAQ runs were used for PGC's identified in multiple runs. “Total”, “Analyzed” and “Panel” represent the sets of PGC's detected in at least one of the 18 samples included in the discovery, detected in at least 2/3 of the AR and NR groups, and identified with significant differential relative concentrations, respectively. Each bar represents the proportion of PGC's within each group identified based on p distinct peptides. (PDF) [file pcbi.1002963.s005.pdf]

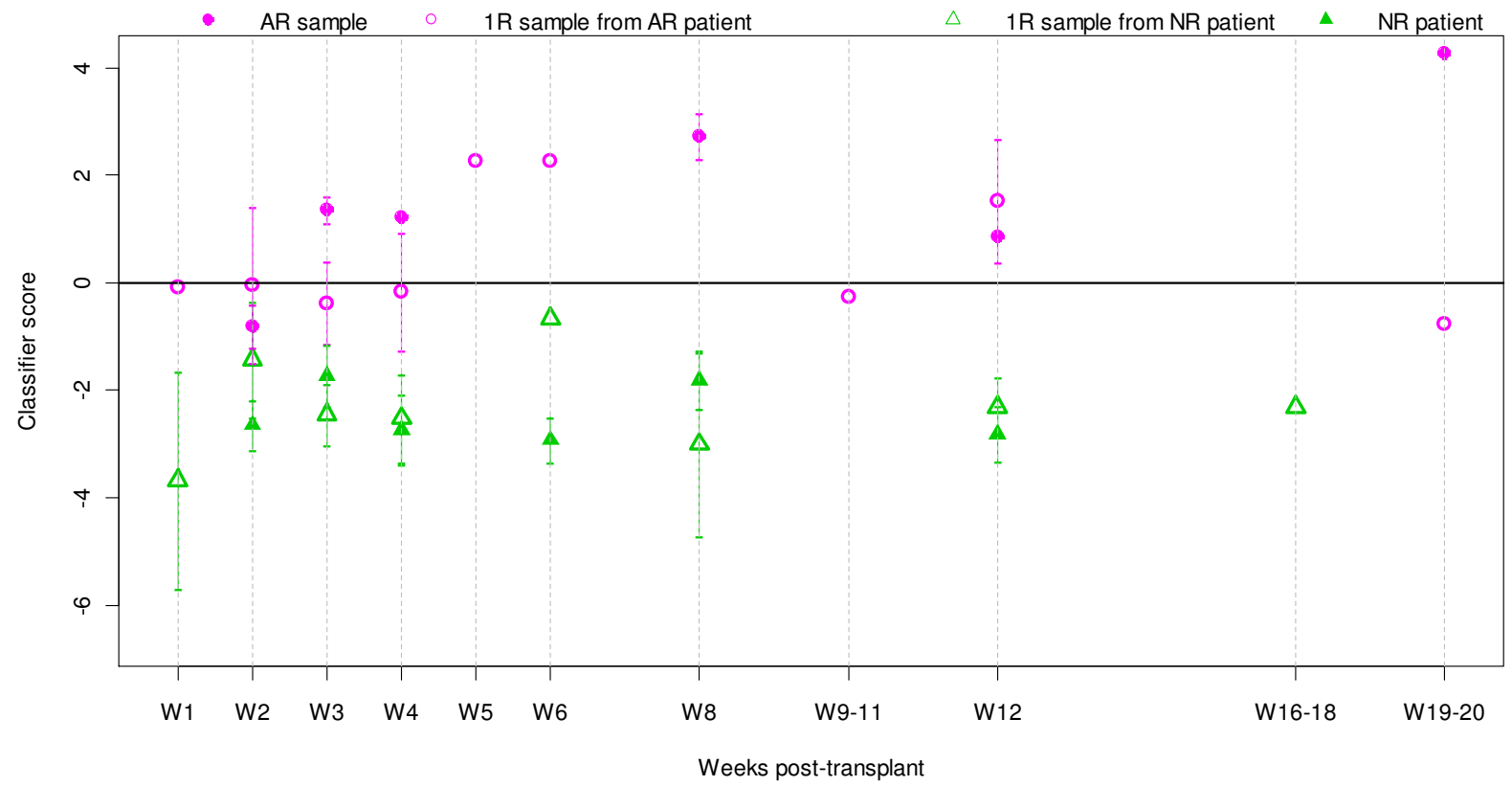

|                     |   |   |    |   |   |   |   |   |   |   |   |    |
|---------------------|---|---|----|---|---|---|---|---|---|---|---|----|
| AR from AR patients | 0 | 2 | 2  | 1 | 0 | 0 | 2 | 0 | 2 | 0 | 1 | 10 |
| 1R from AR patients | 1 | 2 | 5  | 3 | 1 | 1 | 0 | 1 | 3 | 0 | 1 | 18 |
| 1R from NR patients | 2 | 4 | 6  | 5 | 0 | 1 | 2 | 0 | 8 | 1 | 0 | 29 |
| 0R from NR patients | 0 | 9 | 11 | 8 | 0 | 2 | 3 | 0 | 7 | 0 | 0 | 40 |

Supplement: Figure S7 — Classifier score for 1R samples. Average score generated by LDA for all available AR samples (pink solid dot), 1R samples from AR patients (pink open dot), 1R samples from NR patients (green open triangle), and NR samples from NR patients (green solid triangle), at each time point. Standard errors are represented with vertical lines. Sample sizes available at each time point are shown in the table. (PDF) [file pcbi.1002963.s007.pdf]

**A**

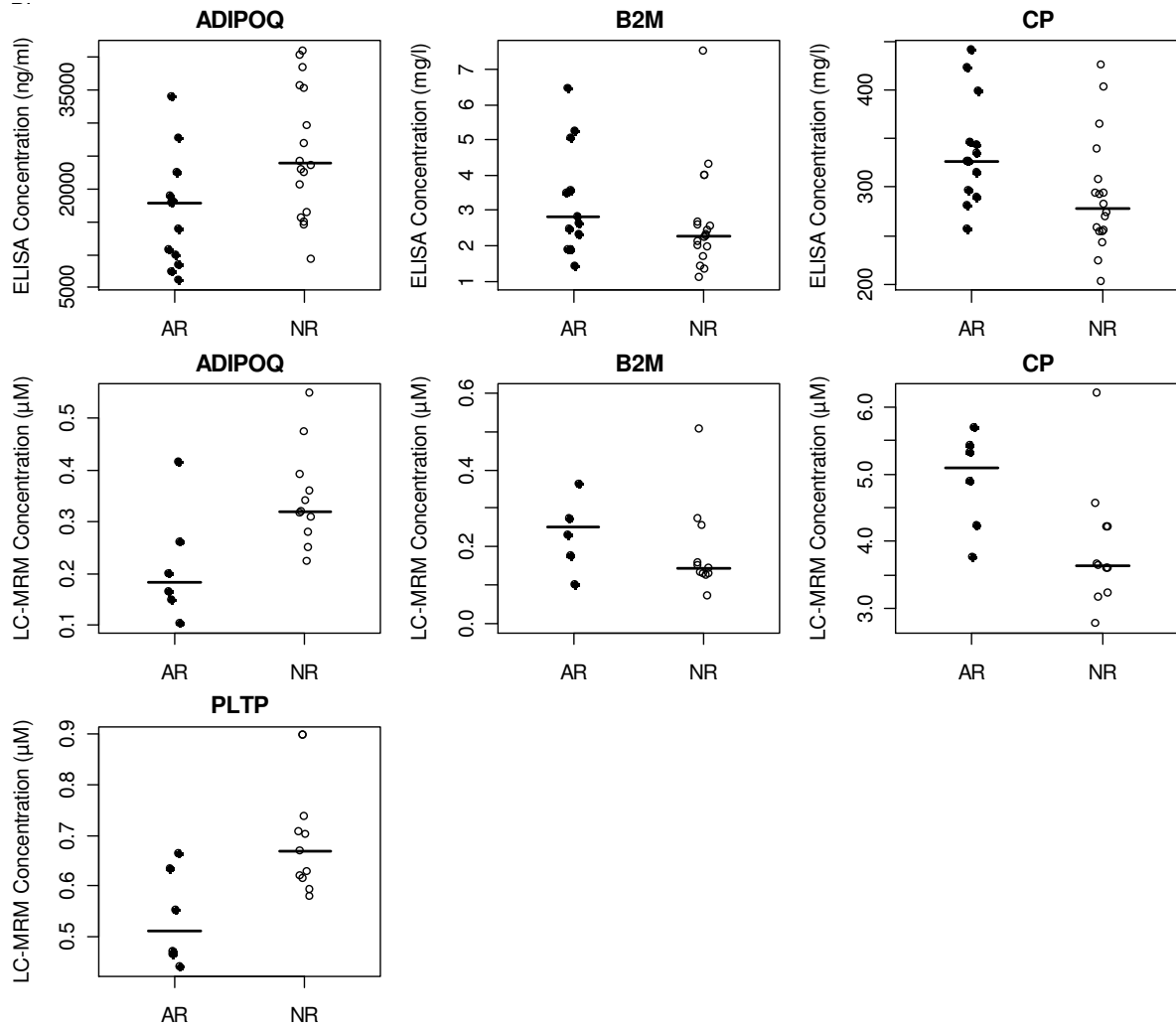

**B**

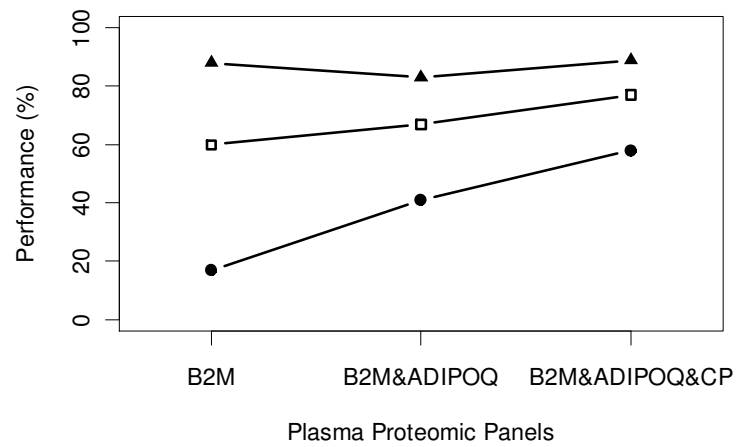

Supplement: Figure S8 — Technical validation. A. Scatter plots of protein concentrations (y-axis) for 13 AR versus 18 NR samples (x-axis) in ELISA/INA, and 6 AR versus 11 NR in MRM-MS for the validated proteins. Median values are represented by horizontal lines within each group. B. Classifiers performance (y-axis) estimated by a cross-validation: Sensitivity (solid dot), specificity (solid triangle), and accuracy (open square) for incremental classifier panels. The x-axis shows three nested classifier panels based on a single marker (B2M), 2 markers (B2M&ADIPOQ) and 3 markers (B2M&ADIPOQ&CP), respectively, measured by ELISA/INA. As F10 and PLTP were not validated in ELISA/INA they were not included in any ELISA/INA-based classifier. (PDF) [file pcbi.1002963.s008.pdf]
