# Supplementary material for: Computational Biomarker Pipeline from Discovery to Clinical Implementation: Plasma Proteomic Biomarkers for Cardiac Transplantation
Source: PLoS Comput Biol. 2013 Apr 4;9(4):e1002963. doi: 10.1371/journal.pcbi.1002963 (PMC3617196; doi:10.1371/journal.pcbi.1002963)

A

| iTRAQ 26 Patients; 108 Samples | ELISA 43 Patients/Samples | MRM 23 Patients/Samples |
|---|---|---|

| Patient | W1 | W2 | W3 | W4 | W5 | W6 | W8 | W9-11 | W12 | W16-18 | W19-20 |
|---|---|---|---|---|---|---|---|---|---|---|---|
| AR1* | | 2R(I) | 1R(I) | 2R(I) | | | 0R(I) | | 2R(I,E,M) | | 1R(I) |
| AR2 | 1R(I) | **2R(I,E)** | 2R(I) | 1R(I) | | | 0R(I) | 0R(I) | 0R(I) | 0R(I) | |
| AR3 | | 0R(I) | 1R(I) | | | | 0R(I) | | **2R(I,E,M)** | | |
| AR4 | | 1R(I) | 1R(I,E,M) | ▲ | | | | | 0R(I) | | |
| AR5 | | 1R(I) | 1R(I) | 0R(I) | | | | 1R(I) | 1R(I) | | **2R(I,E,M)** |
| AR6 | | | 0R(I) | | 1R(I) | 1R(I) | **2R(I,E,M)** | | 1R(I) | | |
| AR7 | | | **3R(I,E,M)** | 1R(I) | | | | | 0R(I) | | |
| AR8 | | | 1R(I) | | | | **2R(I,E,M)** | | 1R(I) | | |
| AR9 | 2R(E) | | | | | | | | | | |
| AR10 | 2R(E) | | | | | | | | | | |
| AR11 | 2R(E) | | | | | | | | | | |
| AR12 | | | 2R(E) | | | | | | | | |
| AR13 | | 2R(E) | | | | | | | | | |
| AR14 | | 2R(E) | | | | | | | | | |
| NR15* | | 0R(I) | 0R(I) | 0R(I) | | | | | 0R(I) | | |
| NR16 | 1R(I) | 0R(I) | 0R(I) | 0R(I) | | | | | **0R(I,E)** | | |
| NR17 | | 0R(I) | 0R(I) | | | 0R(I) | **0R(I,E,M)** | | 1R(I) | | |
| NR18 | | 1R(I) | 0R(I) | | | **0R(I,E,M)** | | | 0R(I) | | |
| NR19 | | 1R(I) | 0R(I) | 0R(I) | | | | | **0R(I,E,M)** | | |
| NR20 | | **0R(I)** | 1R(I,E,M) | | | | | | 1R(I) | 1R(I) | |
| NR21 | 1R(I) | 0R(I) | 0R(I) | **0R(I)** | | | 1R(I,E,M) | | | | |
| NR22 | | | 1R(I) | 1R(I) | | 1R(I) | | | 1R(I,E,M) | | |
| NR23 | | 1R(I) | 1R(I) | | | | | | **0R(I,E,M)** | | |
| NR24 | | **0R(I,E,M)** | | 1R(I) | | | | | 1R(I) | | |
| NR25 | | | **0R(I,E,M)** | 1R(I) | | | | | 0R(I) | | |
| NR26* | | | 0R(I) | 0R(I) | | | | | 1R(I,E,M) | | |
| NR27 | | | 0R(I) | | | | | | **0R(I,E,M)** | | |
| NR28 | | | 1R(I,E,M) | 1R(I) | | | 1R(I) | | | | |
| NR29 | | 1R(I) | 1R(I) | 0R(I) | | | **0R(I,E,M)** | | 1R(I) | | |
| NR30 | | 0R(I) | 0R(I) | 0R(I) | | | **0R(I,E,M)** | | 1R(I) | | |
| NR31 | | 0R(I) | **0R(I,E,M)** | 0R(I) | | | | | 1R(I) | | |
| NR32 | | **0R(I,E,M)** | 1R(I) | 1R(I) | | | | | | | |
| NR33 | | | | | | | | | 1R(E) | | |
| NR34 | | | 0R(E) | | | | | | | | |
| NR35 | | | | | | | | | 0R(E) | | |
| NR36 | | | | | | | 0R(E) | | | | |
| NR37 | | 1R(E) | | | | | | | | | |
| NR38 | | | 1R(E) | | | | | | | | |
| NR39 | | | 0R(E) | | | | | | | | |
| NR40 | | 1R(E) | | | | | | | | | |
| NR41 | | | | | | | 1R(E) | | | | |
| NR42 | | 0R(E) | | | | | | | | | |
| NR43 | | | | | | | 0R(E) | | | | |
| NR44 | | | 1R(E) | | | | | | | | |

B

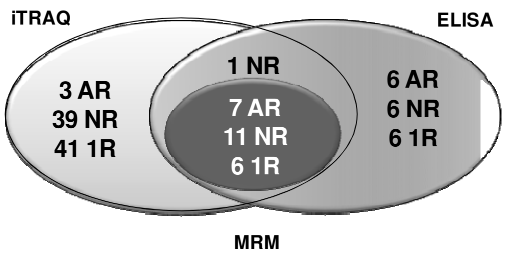

Supplement: Figure S2 — Study design. A. The 3 boxes at the top show the number of patients and samples in the different cohorts. Some of these patient samples were processed by more than one platform to study the correlation between measurements from different technologies. The table shows the ISHLT Grades of rejections and longitudinal distribution of samples processed with iTRAQ (I), ELISA (E) and/or MRM-MS (M). Filled grey cells indicate samples used in the training set of the iTRAQ discovery analysis. Samples graded with an ISHLT Grade ≥2R (multi-foci or diffuse immune cell infiltration with significant associated myocyte damage) were considered to construct the case group (AR, acute rejection). Samples with ISHLT Grade 0R (normal EMB with no evidence of cellular infiltration) were considered for the non-rejection (NR) control group. Mild non-treatable rejections (ISHLT Grade 1R; some cellular infiltrate with limited or absence of myocyte damage; 1R) were only used as test samples. Asterisks indicate patients with additional complications (e.g., prolonged peri-transplant ischemia, infection, second transplant, etc). Black triangle represents AR biopsy with no plasma sample available. B. Overlap of samples in the iTRAQ, the ELISA/INA, and the MRM-MS cohorts. A subset of these samples were included in the training sets of the discovery and corroboration analyses (highlighted cells in panel A of this ). (PDF) [file pcbi.1002963.s002.pdf]
