# Supplementary material for: Computational Biomarker Pipeline from Discovery to Clinical Implementation: Plasma Proteomic Biomarkers for Cardiac Transplantation
Source: PLoS Comput Biol. 2013 Apr 4;9(4):e1002963. doi: 10.1371/journal.pcbi.1002963 (PMC3617196; doi:10.1371/journal.pcbi.1002963)
Supplement: Figure S3 — ProteinPilot's local groups, related protein sequences, and identified peptides. A. Example of the protein group corresponding to β2-microglobulin (B2M) as shown in the protein summaries given by ProteinPilot for three distinct experimental iTRAQ runs (ExpID) in the cardiac biomarker study. The Unused, Total and %Cov are identification quality parameters given by ProteinPilot. Quantitative values corresponding to only one of the 3 ratios is shown (115∶114). B. Aligned protein sequences from the B2M protein group. Peptides identified by Paragon Software within each experimental iTRAQ runs are shown in bold-black fonts. The accession number chosen by Pro Group Algorithm to represent the group in the protein and peptide summaries (top-identifier) is shown in bold-black font. (PDF) [file pcbi.1002963.s003.pdf]

A

ExpID	N	Unused	Total	%Cov	Accession	Name	115:114	PVal 115:114	EF 115:114	...
iTRAQ1	174	4.17	4.17	35.64	IPI:IPI00868938.1	Gene_Symbol=- Beta-2-microglobulin	1.473	0.046	1.459	...
	174	0	4.15	26.05	IPI:IPI00796379.1	Gene_Symbol=B2M B2M protein				
	174	0	2.17	20.97	IPI:IPI00004656.2	Gene_Symbol=B2M Beta-2-microglobulin				
iTRAQ2	152	3.7	3.7	15.84	IPI:IPI00868938.1	Gene_Symbol=- Beta-2-microglobulin	1.290	0.255	3.942	...
	152	0	3.7	13.45	IPI:IPI00796379.1	Gene_Symbol=B2M B2M protein				
	152	0	1.7	4.84	IPI:IPI00004656.2	Gene_Symbol=B2M Beta-2-microglobulin				
iTRAQ3	155	4	4.05	27.73	IPI:IPI00796379.1	Gene_Symbol=B2M B2M protein	1.071	0.725	6.698	...
	155	0	4.05	22.77	IPI:IPI00868938.1	Gene_Symbol=- Beta-2-microglobulin				
	155	0	2	12.90	IPI:IPI00004656.2	Gene_Symbol=B2M Beta-2-microglobulin				

B

iTRAQ experiment 1

IPI00868938.1 MSRSVALAVLALLSLSGLEAIQRTPK**IQVYSR** -------------------------------HPSDIEVDLL 42
IPI00796379.1 MSRSVALAVLALLSLSGLEAIQRTPK**IQVYSR**HPAENGKSNFLNCYVSGFHPSDIEVDLL 60
IPI00004656.2 MSRSVALAVLALLSLSGLEAIQRTPK**IQVYSR**HPAENGKSNFLNCYVSGFHPSDIEVDLL 60

IPI00868938.1 KNGERIEKVEHSDLSFSKDWSFYLLY**YTEFTPTEKDEYACRVNHVTLS -------QPK**IVK**W** 97
IPI00796379.1 KNGERIEKVEHSDLSFSKDWSFYLLY**YTEFTPTEKDEYACRVNHVTLS -------QPK**IVKW 115
IPI00004656.2 KNGERIEKVEHSDLSFSKDWSFYLLY**YTEFTPTEKDEYACR**VNHVHLIIWAIRQPKIVK**W** 120

IPI00868938.1 **DRDM** 101
IPI00796379.1 DRDI 119
IPI00004656.2 **DRDM** 124

iTRAQ experiment 2

IPI00868938.1 MSRSVALAVLALLSLSGLEAIQRTPK**IQVYSR** ------------------------------- HPSDIEVDLL 42
IPI00796379.1 MSRSVALAVLALLSLSGLEAIQRTPK**IQVYSR**HPAENGKSNFLNCYVSGFHPSDIEVDLL 60
IPI00004656.2 MSRSVALAVLALLSLSGLEAIQRTPK**IQVYSR**HPAENGKSNFLNCYVSGFHPSDIEVDLL 60

IPI00868938.1 KNGERIEKVEHSDLSFSKDWSFYLLYYTEFTPTEKDEYACR**VNHVTLS ------ QPK**IVKW 97
IPI00796379.1 KNGERIEKVEHSDLSFSKDWSFYLLYYTEFTPTEKDEYACR**VNHVTLS ------ QPK**IVKW 115
IPI00004656.2 KNGERIEKVEHSDLSFSKDWSFYLLYYTEFTPTEKDEYACRVNHVHLIIWAIRQPKIVKW 120

IPI00868938.1 DRDM 101
IPI00796379.1 DRDI 119
IPI00004656.2 DRDM 124

iTRAQ experiment 3

IPI00868938.1 MSRSVALAVLALLSLSGLEAIQR**TPKIQVYSR** ------------------------------- HPSDIEVDLL 42
IPI00796379.1 MSRSVALAVLALLSLSGLEAIQR**TPKIQVYSRHPAENGK**SNFLNCYVSGFHPSDIEVDLL 60
IPI00004656.2 MSRSVALAVLALLSLSGLEAIQR**TPKIQVYSRHPAENGK**SNFLNCYVSGFHPSDIEVDLL 60

IPI00868938.1 KNGERIEKVEHSDLSFSKDWSFYLLYYTEFTPTEKDEYACR**VNHVTLS ------ QPKIVKW** 97
IPI00796379.1 KNGERIEKVEHSDLSFSKDWSFYLLYYTEFTPTEKDEYACR**VNHVTLS ------ QPKIVKW** 115
IPI00004656.2 KNGERIEKVEHSDLSFSKDWSFYLLYYTEFTPTEKDEYACRVNHVHLIIWAIRQPKIVK**W** 120

IPI00868938.1 **DRD**M 101
IPI00796379.1 **DRD**I 119
IPI00004656.2 **DRD**M 124
