# Supplementary material for: Computational Biomarker Pipeline from Discovery to Clinical Implementation: Plasma Proteomic Biomarkers for Cardiac Transplantation
Source: PLoS Comput Biol. 2013 Apr 4;9(4):e1002963. doi: 10.1371/journal.pcbi.1002963 (PMC3617196; doi:10.1371/journal.pcbi.1002963)
Supplement: Figure S6 — Classification results. Set of AR and NR samples in the test set classified based on the LDA score. Samples used in the discovery and 1R samples were not included in the test set. Biopsy and classifier results are shown in the top-left and bottom-right corners of each cell, respectively. For example, the second week sample (W2) of the first acute rejection patient (AR1) was classified as AR based on the biopsy (top-left) and as a 0R based on the proteomic classifier (bottom-right). Misclassified samples are highlighted with filled bold-font cells. (PDF) [file pcbi.1002963.s006.pdf]

Biopsy / Classifier	*Patient with complications	**misclassified test samples**

Patient ID	W1	W2	W3	W4	W6	W8	W9-11	W12	W16-18	W19-20
AR1*		**AR / NR**		AR / AR		**NR / AR**		AR / AR		
AR2			AR / AR			NR / NR	NR / NR	NR / NR	NR / NR	
AR3		NR / NR				NR / NR				
AR4								**NR / AR**		
AR5				NR / NR						
AR6			**NR / AR**							
AR7								NR / NR		
NR15*		**NR / AR**	**NR / AR**	**NR / AR**				NR / NR		
NR16		NR / NR	NR / NR	NR / NR						
NR17		NR / NR	NR / NR		NR / NR					
NR18			NR / NR					NR / NR		
NR19			**NR / AR**	**NR / AR**						
NR21		NR / NR	NR / NR							
NR25								NR / NR		
NR26*			NR / NR	NR / NR						
NR27			NR / NR							
NR29				NR / NR						
NR30		NR / NR	NR / NR	NR / NR						
NR31		NR / NR		NR / NR						
