# Supplementary material for: Computational Biomarker Pipeline from Discovery to Clinical Implementation: Plasma Proteomic Biomarkers for Cardiac Transplantation
Source: PLoS Comput Biol. 2013 Apr 4;9(4):e1002963. doi: 10.1371/journal.pcbi.1002963 (PMC3617196; doi:10.1371/journal.pcbi.1002963)
Supplement: Table S1 — Demographic characteristics of patients. Numbers in parentheses are percentages unless otherwise stated. (PDF) [file pcbi.1002963.s009.pdf]

Characteristics	Acute rejection	Non-rejection
Subjects	17	27
Mean age (SD)	51 (13)	53 (14)
Female	6 (35%)	3 (11%)
Male	11 (65%)	24 (89%)
Ethnicity		
Caucasian	17 (100%)	24 (89%)
Asian	0 (0%)	1 (4%)
Other	0 (0%)	2 (7%)
Primary disease		
Cardiomyopathy - Ischemic (Coronary Artery Disease)	7 (41%)	13 (48%)
Cardiomyopathy - Idiopathic Dilated	3 (18%)	2 (7%)
Cardiomyopathy - Dilated	5 (29%)	6 (22%)
Cardiomyopathy - Hypertrophic	1 (6%)	0 (0%)
Congenital heart disease	0 (0%)	3 (11%)
Cardiogenic shock	0 (0%)	3 (11%)
Other	1 (6%)	0 (0%)
