# Supplementary material for: Computational Biomarker Pipeline from Discovery to Clinical Implementation: Plasma Proteomic Biomarkers for Cardiac Transplantation
Source: PLoS Comput Biol. 2013 Apr 4;9(4):e1002963. doi: 10.1371/journal.pcbi.1002963 (PMC3617196; doi:10.1371/journal.pcbi.1002963)
Supplement: Table S3 — Corroboration of the discovery results by a different statistical test. Comparison of p values calculated by the robust eBayes test (fifth column) used in the iTRAQ discovery and the Wilcoxon test (sixth column). Tests were based on iTRAQ data for the 5 identified candidate markers (6 AR versus 14 NR samples). (PDF) [file pcbi.1002963.s011.pdf]

PGC	Accession	Protein Name	Gene Symbol	p-value eBayes	p-value Wilcoxon	Fold-Change
6	IPI00017601.1	Ceruloplasmin precursor	CP	0.002	0.008	+1.28
151	IPI00643034.2	Isoform 1 of Phospholipid transfer protein precursor	PLTP	0.003	0.039	-1.56
	IPI00217778.1	Isoform 2 of Phospholipid transfer protein precursor				
	IPI00022733.3	45 kDa protein				
188	IPI00004656.2	Beta-2-microglobulin	B2M	0.004	0.070	+1.46
	IPI00796379.1	B2M protein				
	IPI00868938.1	Beta-2-microglobulin				
84	IPI00019576.1	Coagulation factor X precursor	F10	0.006	0.013	+1.27
	IPI00552633.2	Coagulation factor X				
92	IPI00020019.1	Adiponectin precursor	ADIPOQ	0.007	0.014	-1.31
