# Supplementary material for: Computational Biomarker Pipeline from Discovery to Clinical Implementation: Plasma Proteomic Biomarkers for Cardiac Transplantation
Source: PLoS Comput Biol. 2013 Apr 4;9(4):e1002963. doi: 10.1371/journal.pcbi.1002963 (PMC3617196; doi:10.1371/journal.pcbi.1002963)
Supplement: Table S4 — Quality control parameters of proteomic data. “Unused” represents the median of the Unused ProtScores calculated by ProteinPilot for the top protein within each iTRAQ run protein group. Unused values equal to 2.0 is equivalent to a 99% confidence. Similarly, “Coverage” and “Error factor” represent the median of percent coverage and error factor measures calculated by ProteinPilot for each group in each iTRAQ run. “Peptide count” shows the average of unique peptide counts, excluding miscleavages, used for protein identification and quantitation by ProteinPilot in each iTRAQ run. “Missing AR/NR” shows the number of samples in the rejection (AR) and non-rejection (NR) groups in which each protein group was not detected. “Length” and “pI/molecular mass” contain the number of amino acids in each sequence and the isoelectric point/molecular mass (kDa) for each protein, respectively. aValues in these columns correspond to the PGC and not to a particular protein identifier. (PDF) [file pcbi.1002963.s012.pdf]

PGC	Accession	Unused[a]	Coverage[a]	Error Factor[a]	Peptide Count[a]	Missing AR/0R[a]	Length	pI/molecular mass
6	IPI00017601.1	97.94	55.77	1.08	48.4	0/0	1065	5.44 / 122.22
151	IPI00643034.2	3.58	13.08	2.62	2.6	2/2	493	6.53 / 54.75
	IPI00217778.1						441	6.11 / 49.28
	IPI00022733.3						405	8.69 / 44.85
188	IPI00004656.2	2.25	15.84	3.49	2.1	0/3	124	6.51 / 14.42
	IPI00796379.1						119	6.06 / 13.69
	IPI00868938.1						101	5.88 / 11.75
84	IPI00019576.1	11.64	29.71	1.26	6.7	0/0	488	5.68 / 54.74
	IPI00552633.2						332	5.63 / 37.09
92	IPI00020019.1	6.02	23.36	1.66	3.7	0/1	244	5.42 / 26.42
