# Supplementary material for: Computational Biomarker Pipeline from Discovery to Clinical Implementation: Plasma Proteomic Biomarkers for Cardiac Transplantation
Source: PLoS Comput Biol. 2013 Apr 4;9(4):e1002963. doi: 10.1371/journal.pcbi.1002963 (PMC3617196; doi:10.1371/journal.pcbi.1002963)
Supplement: Table S5 — Confounding factors. The GlobalAncova analysis evaluates if the panel protein levels remain significantly differentiated between the acute rejection (AR) and the non-rejection (NR) groups after adjusting for potential confounding factors. A p value below 0.05 provides evidence of significant differentiation. We use all clinical data available at the time closest to the collection time of the plasma sample measured by iTRAQ. The correlation between the value of potential confounders and the LDA classifier score was evaluated using a Pearson correlation coefficient. The last two columns show the mean and standard deviation (SD) of the clinical variables for the 6 AR samples and 14 NR samples in the discovery cohort. (PDF) [file pcbi.1002963.s013.pdf]

Potential confounders	GlobalAncova *p* value	Correlation with score	Acute rejection Mean (SD)	Non-rejection Mean (SD)
BUN in blood (mmol/L)	0.011	-0.43	14.63 (6.12)	11.64 (5.26)
Creatinine in blood (umol/L)	0.004	-0.42	145.17 (53.83)	125.86 (55.4)
Glomerular Filtration Rate from blood (mL/min)	0.002	0.30	52.17 (30.12)	55.76 (24.41)
Albumin in blood (g/L)	0.005	-0.22	42.67 (2.94)	41.82 (3.71)
Potassium in blood (mmol/L)	0.004	-0.28	4.13 (0.44)	4.05 (0.5)
Glucose in blood (mmol/L)	0.036	-0.35	6.5 (1.99)	6.06 (2.15)
Total Carbon Dioxide in blood (mmol/L)	0.004	0.47	25.17 (2.32)	26.14 (1.51)
Protein in blood (g/L)	0.007	-0.08	68.33 (4.27)	66.3 (5.01)
Magnesium in blood (mmol/L)	0.004	-0.35	0.78 (0.11)	0.76 (0.21)
Uric acid in blood (umol/L)	0.013	-0.38	487 (141.15)	329.7 (83.11)
Neutrophil Number in blood (xA9/L)	0.009	-0.02	6.77 (4.58)	6.56 (4.56)
Hemaglobin in blood (g/L)	0.020	0.42	110.17 (21.55)	120.57 (14.97)
Whole Blood Count(xA9/L)	0.009	0.00	9.28 (4.99)	9.17 (5.68)
Platelet in blood (xA9/L)	0.007	0.06	268.33 (69.53)	280.14 (71.65)
Mycophonelate Mofetil daily dose (mg)	0.007	-0.38	2250 (524.4)	1821.43 (540.91)
Cyclosporine daily dose (mg)	0.005	-0.18	175 (161.25)	167.86 (195.48)
Prednisone daily dose (mg)	0.013	0.05	10.83 (8.01)	11.79 (6.08)
Tacrolimus daily dose (mg)	0.014	0.41	1.67 (4.08)	4.00 (5.22)
Systolic blood pressure (mmHg)	0.015	0.34	133.67 (15.71)	122.00 (18.13)
Weight (kg)	0.008	-0.17	74.38 (17.89)	76.76 (29.88)
Diastolic blood pressure (mmHg)	0.012	0.07	74.17 (10.23)	71.92 (22.00)
