# Supplementary material for: Computational Biomarker Pipeline from Discovery to Clinical Implementation: Plasma Proteomic Biomarkers for Cardiac Transplantation
Source: PLoS Comput Biol. 2013 Apr 4;9(4):e1002963. doi: 10.1371/journal.pcbi.1002963 (PMC3617196; doi:10.1371/journal.pcbi.1002963)
Supplement: Text S1 — A detailed description of sample selection criteria, plasma collection, depletion, trypsin digestion, iTRAQ labeling, 2D-LC chromatography, mass spectrometry, data processing procedures and analyses are given in this supporting material. (PDF) [file pcbi.1002963.s014.pdf]

SUPPORTING MATERIAL

Computational biomarker pipeline from discovery to clinical implementation: plasma proteomic biomarkers for cardiac transplantation

A detailed description of sample selection criteria, plasma collection, depletion, trypsin digestion, iTRAQ labeling, 2D-LC chromatography, mass spectrometry, data processing procedures and analyses are given in this supporting material.

Patients

A prospective, longitudinal study, approved by the Human Research Ethics Board of the University of British Columbia, was conducted on 63 patients, with signed consent, who received a cardiac transplant at St. Paul's Hospital, Vancouver, British Columbia between March 2005 and February 2008. A total of 708 plasma samples from the 63 enrolled patients were collected by protocol prior *to* transplant and serially at 14 time points for up to 3 years post-transplant, and at times of suspected rejection. Of these 63 patients, 44 were included in the acute rejection cohort according to their type of rejection or matched time points. All AR samples were collected within 2 days of biopsy-proven rejection episodes.

In general, transplant patients received basilimax induction followed by standard triple immunosuppressive therapy consisting of cyclosporine, prednisone and mycophenolate mofetil. Basilimax induction was used as a standard protocol. Patients with acute rejection received appropriate treatments for acute rejection. All patients were prescribed standard lipid therapy (in general, pravastatin) after transplant.

Sample Size Calculation

A power calculation analysis was used to determine statistically the number of samples needed in the discovery using iTRAQ and the technical validation using ELISA/INA (Figure S1A and S1B, respectively) [1,2].

First, the analysis for the discovery was based on an estimated standard deviation of 0.25 for logged relative protein levels measured by iTRAQ. This variation was initially estimated based on a validation experiment run by the Biomarkers in Transplantation group (unpublished data) and it is consistent with the estimates published by Song et al. based on human plasma samples of osteoarthritis patients [3]. The red, green, and blue curves illustrate the sample size required (x-axis) to test a significant (two-sided alpha=0.01) difference between the acute rejection (AR) and the non-rejection (NR) groups with different power levels (y-axis) if the observed fold-changes (ratio of means of relative protein levels between the case and control groups) are 1.15, 1.2, and 1.3, respectively (equivalent to effect sizes of 1.0, 1.5, and 2.0 in logarithm base 2 scale, respectively). In this analysis it was assumed that the control group is twice as large as the case group. Based on these results, a *t*-test can discriminate a fold-change of 1.2 using less than 10 AR patients and 20 NR patients with 80% power and a type I error rate of 1% (Figure S1A).

Second, the analysis for the validation was based on the coefficient of variations and fold-changes observed in previous ELISA/INA pilot data from this study (right table in Figure S1B). The solid, dotted, and dashed curves illustrate the sample size required (x-axis) to test a significant (two-sided alpha=0.05) difference between the AR and the NR groups with different power levels (y-axis) if the observed coefficient of variations and fold-changes are equal to those observed for ADIPOQ, CP, and B2M, respectively (Figure S1B). These curves show that a test to discriminate between the two groups can be performed using about 15 patients per group and still achieve 80% power with a type I error rate of 5%. We note that although replicates were not considered in the power calculation, these were used in the analysis of

ELISA/INA data increasing the resulting power of the tests. Since FactorX had a very low fold-change without clinical utility, it was not included in the calculation.

Sample Selection Criteria

Samples collected from the 44 patients in the acute rejection cohort at different time points were processed by iTRAQ, ELISA/INA, and/or MRM-MS (Figure S2A). Demographic characteristics of the patients included in this study are summarized in Table S1.

To ensure a rigorous case-control analysis in the iTRAQ discovery study, several sample selection criteria were used to construct a discovery cohort (training set) from all available iTRAQ samples (Figures S2A and S2B): *i*) select one sample per patient to maintain usual assumptions of independence between samples in statistical tests; *ii*) only include non-rejection (NR) samples collected from patients with no AR episode within first 6 months post-TX (NR patients) and the first time point of AR from patients with AR episode within first 6 months post-TX (AR patients); *iii*) do not select patients who did not respond to acute rejection treatment and/or had major/multiple complications within the first 6 months post-transplant (i.e., including prolonged peri-transplant ischemia, infection, second transplant, etc); *iv*) do not select NR samples which were taken during an acute rejection treatment. On average, two NR samples were mapped to each AR sample, collected at approximately matching time points. Figure S2A shows which samples were selected for the (training) discovery set (highlighted cells). All iTRAQ samples not included in the training set were included in the test set for an initial validation based on iTRAQ data.

For the ELISA analysis, single samples from patients enrolled later in the study that were not part of the iTRAQ cohort were selected to extend the original discovery cohort (Figure S2). The 18 new samples were selected to approximately match time points of samples in the discovery cohort (labels 0R(E), 1R(E), and 2R(E) in Figure S2A).

Almost all samples from the discovery set were processed by our developed MRM-MS assay depending on remaining sample material (Figure S2B). All common samples among the different platforms (Figure S2 and Table 1 in the main text) were used to study the correlation among protein levels measured by iTRAQ, ELISA/INA, and MRM-MS. Further details on the resulting study cohorts are given in the Results section.

iTRAQ Sample and Data Processing

Peripheral blood samples were drawn into EDTA tubes and stored on ice. Plasma was separated and stored at -80 °C. Plasma samples were depleted of the 14 most abundant proteins (albumin, fibrinogen, transferrin, IgG, IgA, IgM, haptoglobin, α_2-macroglobulin, α_1-acid glycoprotein, α_1-antitrypsin, apolipoprotein A-I, apolipoprotein A-II, complement C3, and apolipoprotein B) by immunoaffinity chromatography (GenWay Biotech, San Diego, CA), trypsin digested and labeled (Applied Biosystems; Foster City, CA). Trypsin peptides from a common pool of plasma from 16 healthy individuals were labeled with iTRAQ reagent 114 and peptides from 3 patient samples were randomly labeled with reagents 115, 116 and 117. Labeled peptides were pooled, separated by strong cation exchange chromatography (PolyLC Inc., Columbia, MD USA), followed by reverse phase chromatography (Michrom Bioresources Inc., Auburn, CA USA) and spotted directly onto 384 spot MALDI ABI 4800 plates with 4 plates per experiment. Spotted peptides were analyzed by a 4800 MALDI TOF/TOF analyzer (Applied Biosystems; Foster City, CA), and MS/MS data was processed using ProteinPilot software v2.0 (Applied Biosystems) with the integrated new Paragon Search Algorithm [4] (Applied Biosystems) and Pro Group Algorithm. Database searching was performed against the International Protein Index (IPI HUMAN v3.39 database, 69731 entries) [5]. The precursor tolerance was set to 150 ppm, and the iTRAQ fragment tolerance was set to 0.2 Da. Identification parameters were set for trypsin cleavages and cysteine alkylation by methyl methanethiosulfonate with special factors set at urea denaturation and an identification focus on

biological modifications. Features such as modifications, substitutions, and number of missed cleavages allowed are modeled with probabilities by Paragon algorithm and thus are not limited to a fixed value [4]. The detected protein threshold used was Unused ProtScore greater than 0.70 (equivalent to an 80.0% CI) to maximize protein coverage from multiple experiments.

Protein Groups

Based on the list of observed peptides, ProteinPilot assembled the data into protein groups to remove redundancies and comparative quantitations within each iTRAQ run (local). In general, these protein groups consist of more than one related protein identities potentially present in the sample. Figure S3A shows the local protein groups (defined as protein IDs with equal rank N) created by ProteinPilot in three experimental iTRAQ runs from the cardiac biomarker study for one of the proteins in the panel, β_2-microglobulin (B2M). While the same three protein identifiers are in the three groups, they have been placed in different order depending on the list of identified peptides (in bold-black font in Figure S3B). This example shows the challenge of identifying proteins from the lists of identified peptides and the similarity, often observed, among protein sequences within a local group.

In the first experiment, for example, the identification of WDRDM provided additional evidence towards the identification of the first protein ID (IPI00868938.1 in bold-black font in Figure S3B), thus this was selected by Pro Group Algorithm as the top-identifier to represent the group in that experiment (Figure S3A). However, in the same protein ID represents the group in the second experiment, even when there is not additional evidence towards it, i.e., both IPI00868938.1 and IPI00796379.1 are “indistinguishable” based on the list of identified peptides [6]. In the third experiment, the identification of HPAENGK and IVK made the second protein ID the top-identifier of the group (IPI00796379.1 in bold-black font in Figure S3B). In general, similar lists of identified peptides can result in protein groups with different top-proteins

in distinct experimental runs. Noteworthy, in all experiments there was not sufficient evidence to determine if only one or all protein identifications were originally present in the analyzed samples. Thus, to make a comprehensive exploration of the data one should compare protein groups, instead of single protein identities, across multiple experimental runs.

Most relative peptide levels used to identify the group were aggregated by ProteinPilot using weighted geometric means to estimate the relative protein levels (ratios of labels 115, 116 and 117 relative to 114, respectively) for each protein group (Figure S3A). To ensure the comparison of related proteins belonging to similar groups across multiple experiments, a protein group code (PGC) was assigned to all proteins in connected groups by an in-house algorithm, called Protein Group Code Algorithm (PGCA). For the example outlined in the Figure S3, the assigned PGC was 188. This code was assigned to the 3 groups shown in the example, as well as to connected groups from other runs, and it was used to link the data related to this protein from all samples in the study. Additional details on quantitative measures related to Protein Pilot and PGCA have been previously described [7].

Multiplex MRM-MS Assay Development

Potential proteotypic peptides for each of the 5 target proteins were selected based on data obtained from the Peptide Atlas database. Rules of selection were based upon peptide length and hydrophobicity. Additionally, peptides that were likely to digest incompletely or that contained residues that are commonly subject to chemical modifications such as oxidation were avoided. For these analyses, MRM ion pairs for 16 target peptides were selected, and their sensitivities were optimized as previously described [8,9]. The precursor/product ion pairs used for these experiments and their retention times are listed in Table S2. The overall multiplexed MRM assay monitored 174 ion pairs with an analysis time of 60 min.

All reagents were ACS grade or better. All solvents used, including water, were LC/MS grade. Stable isotopically-labeled peptide standards (SIS peptides) were synthesized using

Fmoc chemistry with isotopically- labeled amino acids, [$^{13}C_6$]Lys or [$^{13}C_6{}^{15}N_4$]Arg. The purity of the HPLC-purified peptides was determined by capillary zone electrophoresis (CZE). The absolute concentrations of each synthetic SIS peptide were determined by amino acid analysis. These absolute concentrations were adjusted by the percent purity of each synthetic peptide as determined by CZE.

Plasma digestion and MRM-MS analysis

Plasma tryptic digests were prepared as previously described [10]. Briefly, following overnight digestion with trypsin, the stable isotope internal standards were added to the solution and the entire volume was concentrated and de-salted by solid phase extraction. For the on-line MRM-MS analysis an AB/MDS Sciex 4000 QTRAP with a nano-electrospray ionization source was used [10].

All MRM data was processed using MultiQuant 1.1 (Applied Biosystems) with the MQL algorithm for peak integration. A 2-min retention time window, with “report largest peak” enabled and a 3-point smooth with a peak-splitting factor of 2, was used. The default MultiQuant values for noise percentage and baseline subtraction window were used. All data was manually inspected to ensure correct peak detection and accurate integration. Linear regression of all calibration curves was performed using a standard 1/x (x=concentration ratio) weighting option to aid in covering a wide dynamic range.

Quality control

Depletion. As a first step in most quantitative plasma proteomic studies, typical protocols utilize a depletion method to remove the most abundant proteins. This step is of fundamental importance to partially decrease the large dynamic range of plasma proteins and thus enhance the detection sensitivity in plasma. Although the efficiency of different depletion methods is well documented, it is also important to determine their reproducibility and applicability in large-scale

plasma proteomic studies. Qualitative reproducibility of depletion was assessed by tracking the number of iTRAQ labeled peptides from 9 of the 14 depleted plasma proteins that remained in the flow-through fractions from the affinity chromatography step [11].

Figure S4 illustrates the proportion of the total peptides per depleted protein remaining, on average, in 6 and 14 iTRAQ runs containing results from the AR and NR samples, respectively, in our discovery. Only two samples from the discovery (one AR and one NR) were processed in the same iTRAQ run. Peptide counts from this run were included in both AR and NR groups. All other discovery samples were processed in separate runs. The proportions of the total peptides identified in our samples were relatively constant for all proteins showing that the affinity column's consistency was acceptable from run to run with no distinct pattern between AR and NR groups. On average, the remaining peptides constitute less than 14% of the total peptides per protein with similar amounts of depletion between AR and NR groups (Figure S4). Proteolytic fragments of these proteins, such as fibrinogen fragments, may have been present in the plasma and were not removed by depleting dappling antibodies as they did not contain the appropriate epitopes. In summary, we conclude that results indicate that the depletion was sufficiently reproducible in acquiring homogeneous fractions of depleted plasma with minimal interference from high-abundant proteins.

Proteomic Data. As stated in the Paris Consensus, the general guideline for proteomic data publication (www.mcponline.org), proteins identified by a single peptide should not be considered unless they had a high enough ion score to produce a confident interval (CI) of at least 99.5% and were recurring throughout all the runs of the experiment. However, to maximize protein coverage from multiple experiments, proteins were pre-filtered based on recurrence of identification instead of CI threshold or peptide counts within each experimental run. The resulting lists of protein identities were nevertheless assessed by examining peptide counts of

the analyzed protein groups and the “Unused Protein Score” parameter (equivalent to CI, i.e., Unused>2.0 is equivalent to CI>99%).

Figure S5 illustrates the proportion of PGC’s identified using different peptide counts (*p*). As peptide counts used to identify each PGC differ run to run, average peptide counts across iTRAQ runs were used for those PGC’s identified in multiple runs. The majority of the proteins identified based on only one peptide were not identified in most of iTRAQ runs so they were pre-filtered once the data of the selected samples is assembled and were not further analyzed. As a result, 98% of the 127 analyzed PGC’s were identified based on 2 or more peptides and 60% based on more than 10 distinct peptides, respectively (Figure S5), demonstrating a strong identification of analyzed proteins.

Robust eBayes [12] tests identified a panel of 5 of the 127 analyzed PGC’s with differential relative concentrations between AR and NR ($p < 0.01$). Results were corroborated by the Wilcoxon test showing that 4 of these 5 PGC’s (CP, PLTP, F10, and ADIPOQ) were differentially concentrated in AR compared to NR (*p* value<0.05, Table S3). Figure S5 shows that all these 5 PGC’s were identified based on 2 or more distinct peptide sequences. Other quality parameters obtained from ProteinPilot for these 5 PGC’s were also examined and are reported in Table S4. The average “Coverage” (percentage of total protein sequence covered by identified peptides) was 27. The “Unused” score was, on average, 24 and the error factor was 2 demonstrating a strong confidence on the identified protein identities and their quantitative levels (Table S4).

Classifier score for samples not included in the discovery

A single score was generated by a classifier built based on the iTRAQ relative levels of the 5 PGC’s in the panel using Linear Discriminant Analysis (LDA) [13] for the 20 discovery samples (training set). This classifier was then used to calculate a score and classify the AR and NR samples not included in the discovery analysis (4 AR and 37 NR). Figure S6

summarizes the biopsy-based classification (top-left) *versus* the proteomic-based classification (bottom-right) showing that 3 out the 4 AR and 29 out of the 37 NR tested samples were correctly classified (non-highlighted cells). Similar results were obtained if only a single test sample per patient was selected.

Similarly, a classifier score was calculated for all AR and NR samples (from NR patients) available at each time point. Figure 2 in the main text illustrates the classifier score across time. Figure S7 includes 1R samples from AR and NR patients. Average scores of 1R samples from NR patients were in general not significantly separated from NR samples, while those from AR patients were closer to AR scores. Inference on within group differences, for example week 1 (W1) vs. week 2 (W2) of AR group, requires an analysis that takes into account the within group correlation (i.e., samples from the same patient may be present at different time points). Means and error bars can be used to assess differences of the score between groups at any one time point (samples at each time point are independent within and between groups).

Cross-Validation and Performance Measures

If an independent test set is not available, the classification performance of a biomarkers classifier can be estimated using cross-validation. A stratified 6-fold cross-validation was used in this study. Briefly, the AR and the NR groups are randomly divided into 6 parts and one part from each group are randomly combined to form a fold. In each run of the cross-validation, one fold (test set) is left out and the remaining 5 folds (training set) are used to develop a classifier using LDA. The resulting classifier is used to classify samples in the test fold. The process is repeated until all folds are tested and the overall performance is estimated averaging over that in the 6 folds.

Because the estimated performance depends on the random partitioning of the data, the cross-validation was repeated 100 times (similar results were obtained with more number of iterations) and median performance measurements were calculated.

The performances of different classifiers were evaluated by the sensitivity, specificity and the area under the receiver operating curve (AUC). The sensitivity measures the probability that a case sample (AR in this case) is correctly classified by the classifier. The specificity measures the probability that a control sample (NR in this case) is correctly classified by the classifier. The accuracy is the proportion of correctly classified samples. The receiver operating curve (ROC) illustrates the true positive rate (sensitivity) *versus* the false positive rate (1-specificity) for all decision thresholds (score or probability cut-off). The area under the receiver operating curve (AUC) is equal to the probability that a randomly chosen case sample will have a higher classifier score than a randomly chosen control sample.

ELISA Validation

A total of 43 patient samples were processed by ELISA/INA (Figures S2A and S2B). Of these 43 samples, 18 samples correspond to additional patients that were not part of the iTRAQ discovery study. A total of 3 out of 4 proteins validated by ELISA/INA (B2M, ADIPOQ, and CP) demonstrated differential protein levels in AR *versus* NR (p value<0.05) with relative levels in the same direction (up or down) as iTRAQ results (Fig. 4a in the main text and Figure S8A). Results from the ELISA/INA data provided corroboration of the iTRAQ findings, as well as additional validation in 12 new patients in a different platform than iTRAQ (Figures S2A and 2B).

Although these assays will not be used to complete the validation stage, different performance measures were evaluated for different classifiers, built using LDA and sequentially incorporating the ELISA/INA measurements from each corroborated protein. As for the MRM-MS validation, the sensitivity, specificity, accuracy, and AUC of a classifier score based on multiple markers improved compared to those of classifier scores based on a single marker. Results demonstrate that the sensitivity can improve from 16% for a classifier based only on B2M, often measured in transplantation, to almost 60% for a classifier based on a 3-protein

panel (B2M&ADIPOQ&CP) (Figure S8B). The specificity estimates were high for all classifiers and the AUC improved from 0.37 to 0.76. As ELISA/INA assays were not available for PLTP and iTRAQ results from F10 were not corroborated, these two proteins were not included in any ELISA/INA based classifier.

References

1. Van Belle G., and Martin D. C. (1993) Sample size as a function of coefficient of variation and ratio of means. *The American Statistician* **47**, 165-167.
2. Cohen, J. (1988). Statistical power analysis for the behavioral sciences (2nd ed.). Hillsdale, NJ: Lawrence Erlbaum.
3. Song X, Bandow J, Sherman J, Baker JD, Brown PW, et al. (2008) iTRAQ experimental design for plasma biomarker discovery. J Proteome Res 7: 2952-2958.
4. Shilov, I. V., Seymour, S. L., Patel, A. A., Loboda, A., Tang, W. H., Keating, S. P., Hunter, C. L., Nuwaysir, L. M., and Schaeffer, D. A. (2007) The Paragon Algorithm, a next generation search engine that uses sequence temperature values and feature probabilities to identify peptides from tandem mass spectra. *Mol Cell Proteomics* **6**, 1638-1655.
5. Kersey, P.J. et al. (2004) The International Protein Index: an integrated database for proteomics experiments. *Proteomics* **4**, 1985-1988.
6. Nesvizhskii AI, Aebersold R (2005) Interpretation of shotgun proteomic data: the protein inference problem. Mol Cell Proteomics 4: 1419-1440.
7. Cohen Freue, G. et al. (2010) Proteomic signatures in plasma during early acute renal allograft rejection. *Mol Cell Proteomics* **9**, 1954-1967.
8. Kuzyk, M.A., Parker, C.E. & Borchers, C.H. (2010) Methods in Molecular Biology, Vol. in press. Eds. A. Ivanov & A. Lazarev. Humana Press.

9. Kuzyk, M. et al. (2009) MRM-based, Multiplexed, Absolute Quantitation of 45 proteins in human plasma. *Molecular and Cellular Proteomics* **8**, 1860-1877.

10. Kuzyk, M.A. et al. (2009) A comparison of MS/MS-based, stable-isotope-labeled, quantitation performance on ESI-quadrupole TOF and MALDI-TOF/TOF mass spectrometers. *Proteomics* **9**, 3328-3340.

11. Zhang B., VerBerkmoes N. C., Langston M. A., Uberbacher E., Hettich R. L., and Samatova N. F. (2006) Detecting differential and correlated protein expression in label-free shotgun proteomics. *J. Proteome Res.* **5**, 2909 -2918.

12. Smyth, G.K. (2004) Linear models and empirical bayes methods for assessing differential expression in microarray experiments. *Stat Appl Genet Mol Biol* **3**, Article3.

13. Hastie, et al. (2001) Elements of Statistical Learning: Data Mining, Inference, and Prediction. Springer-Verlag, New York.
